# Supplementary figures and images for: An essential periplasmic protein coordinates lipid trafficking and is required for asymmetric polar growth in mycobacteria
Source: eLife. 2022 Nov 8;11:e80395. doi: 10.7554/eLife.80395 (PMC9678360; doi:10.7554/eLife.80395)

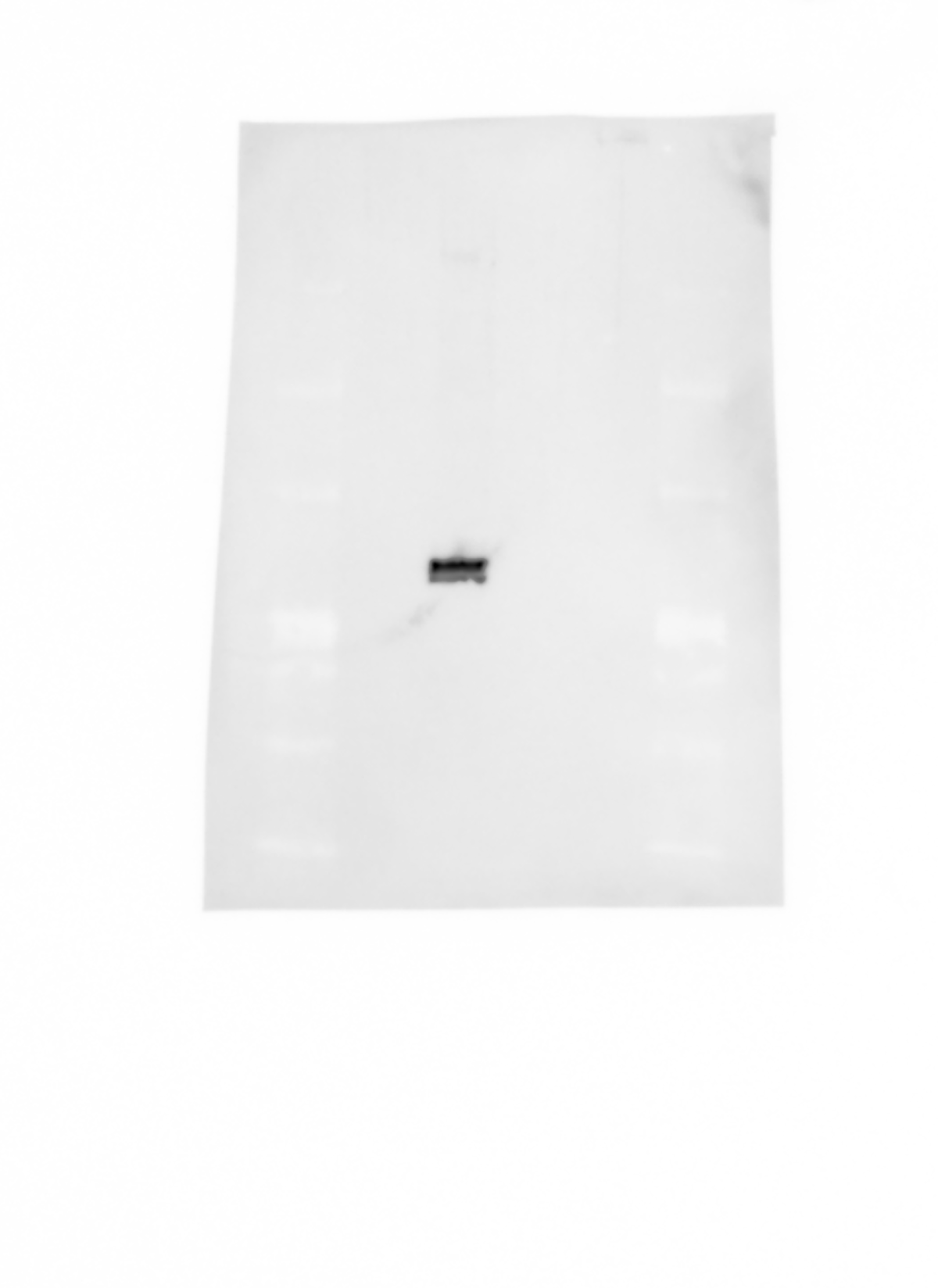

Supplement: Figure 2—source data 1. [file elife-80395-fig2-data1.zip › Figure 2 - source data 1.tif]

Strains expressing:

PgfA-FLAG+ PgfA-FLAG  
MmpL3-msfGFP

PgfA-FLAG

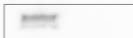

Supplement: Figure 2—source data 2. [file elife-80395-fig2-data2.zip › Figure 2 - source data 2.pdf]

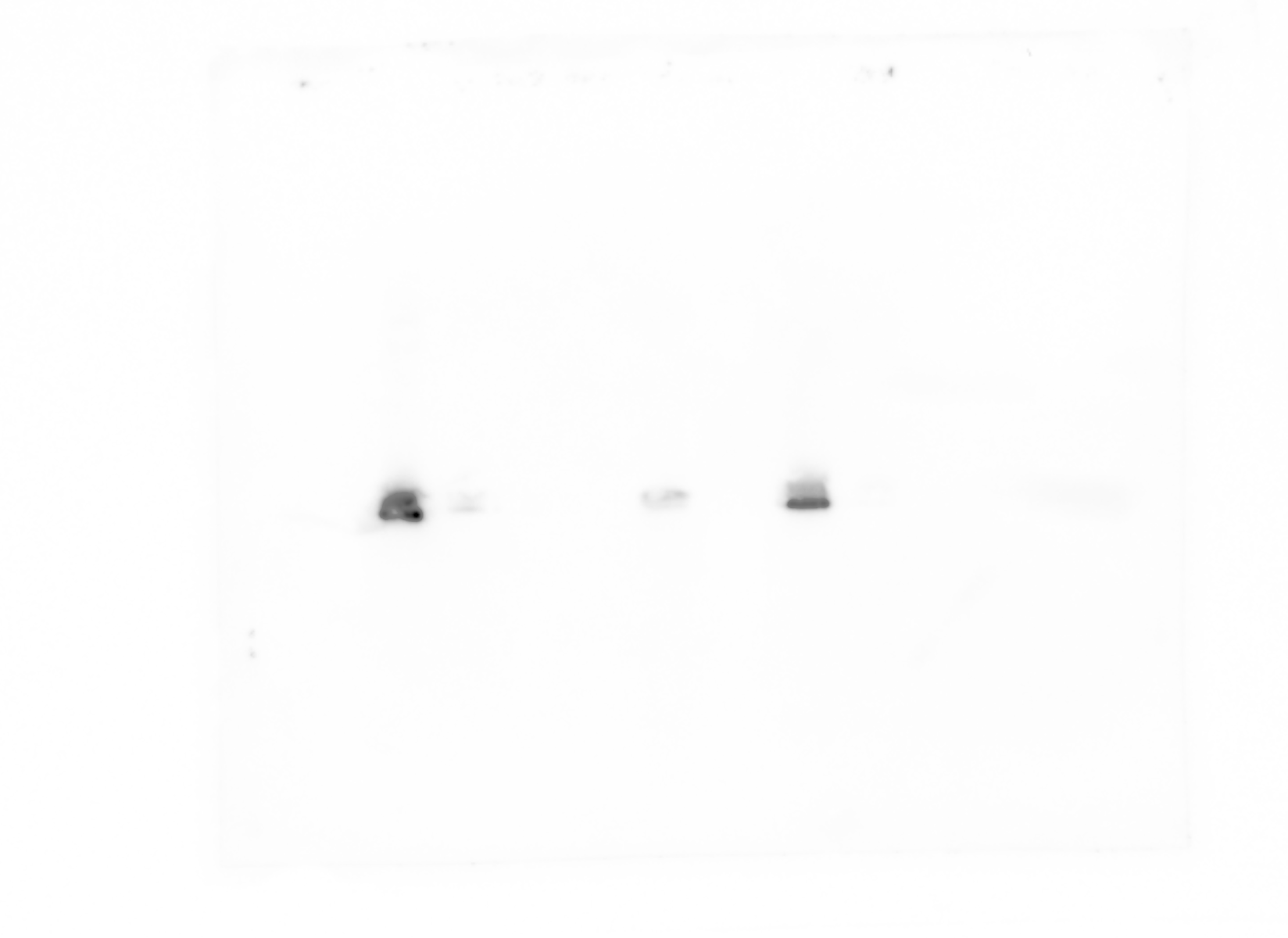

Supplement: Figure 2—figure supplement 2—source data 1. [file elife-80395-fig2-figsupp2-data1.zip › Figure 2 - figure supplement 2 - source data 1.tif]

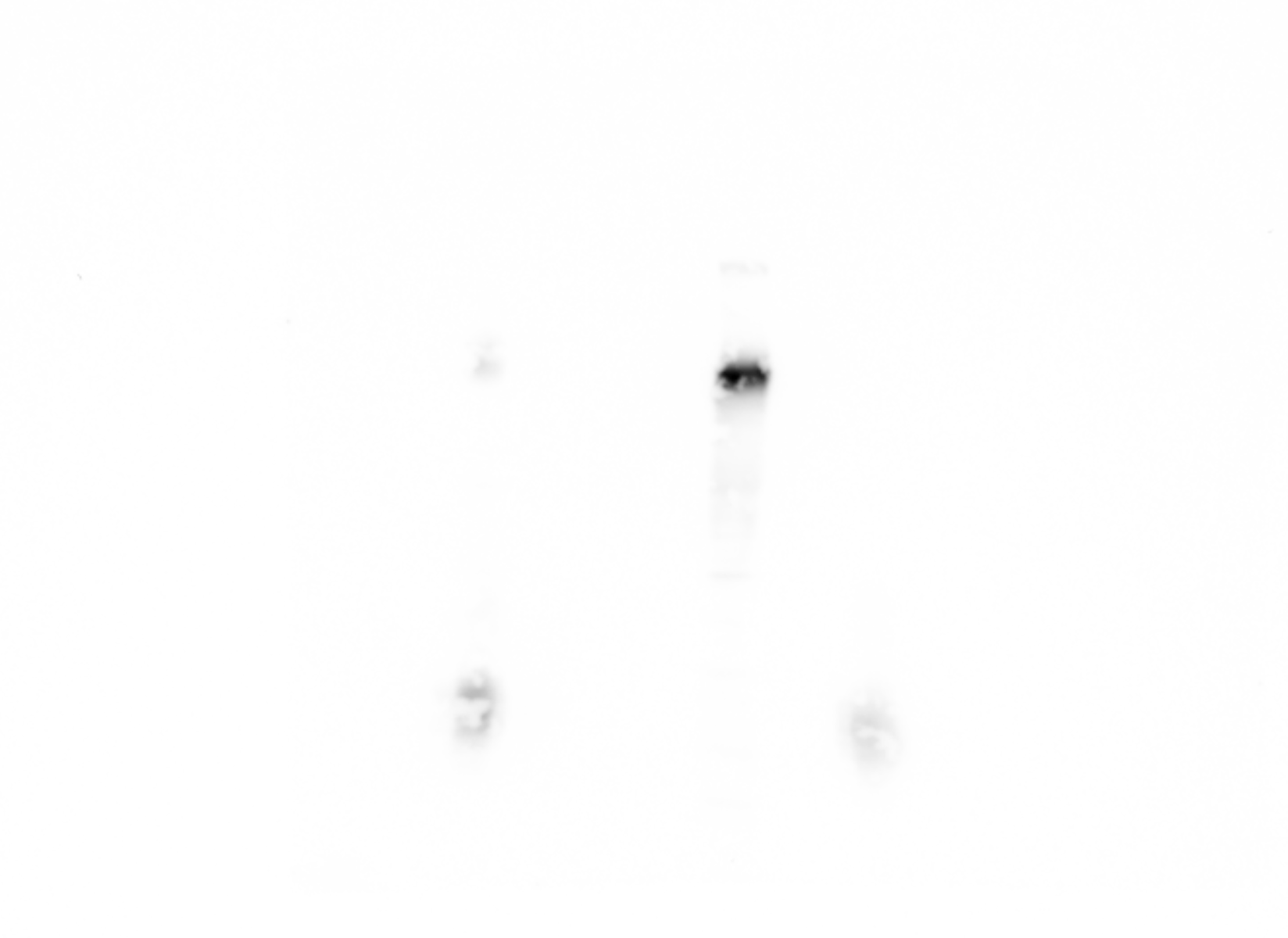

Supplement: Figure 2—figure supplement 2—source data 3. [file elife-80395-fig2-figsupp2-data3.zip › Figure 2 - figure supplement 2 - source data 3.tif]

PgfA-3xFLAG+  
MmpL3-msfGFP

PgfA-3xFLAG

L

washes

E

L

washes

E

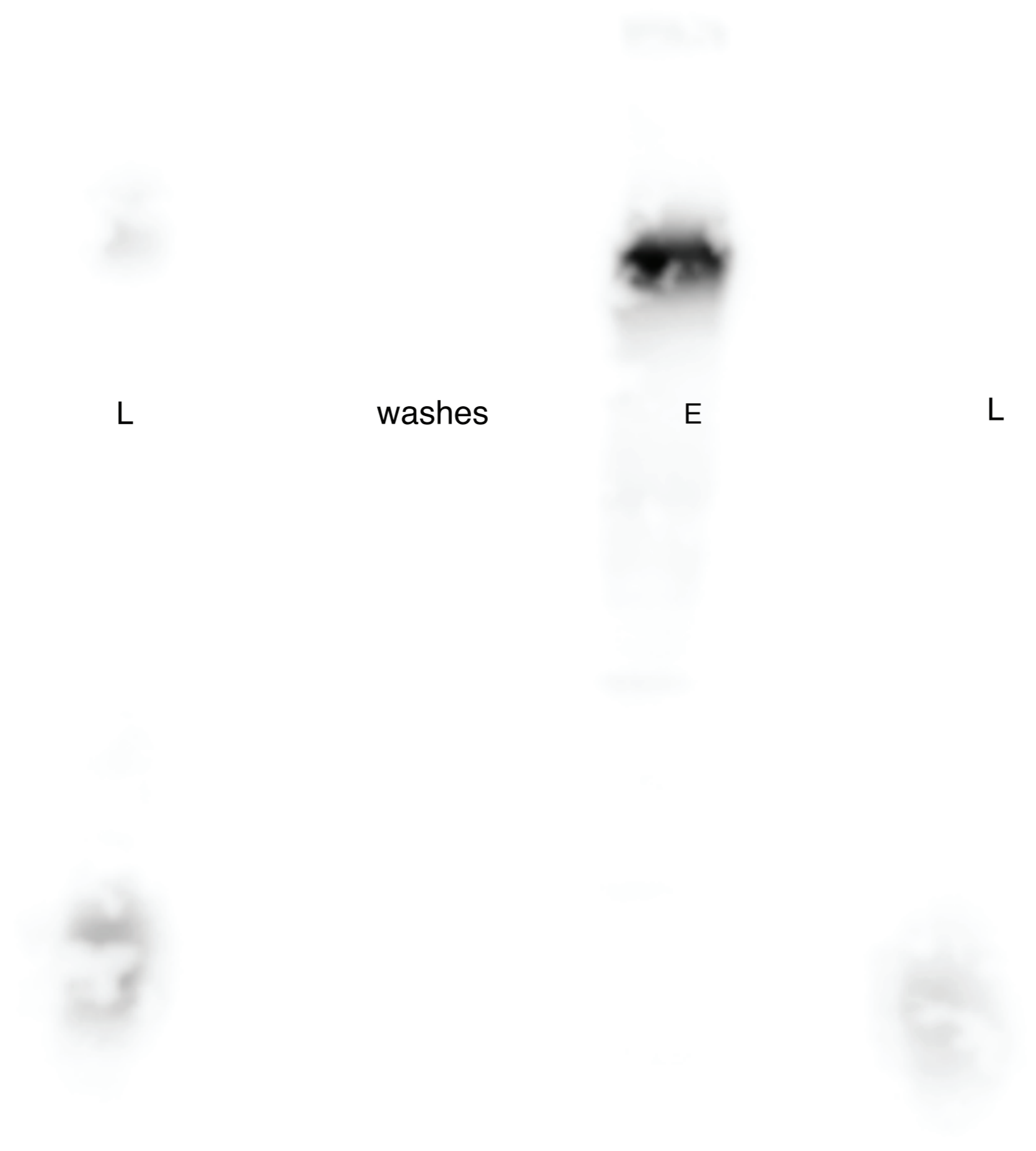

Supplement: Figure 2—figure supplement 2—source data 4. [file elife-80395-fig2-figsupp2-data4.zip › Figure 2 - figure supplement 2 - source data 4.pdf]

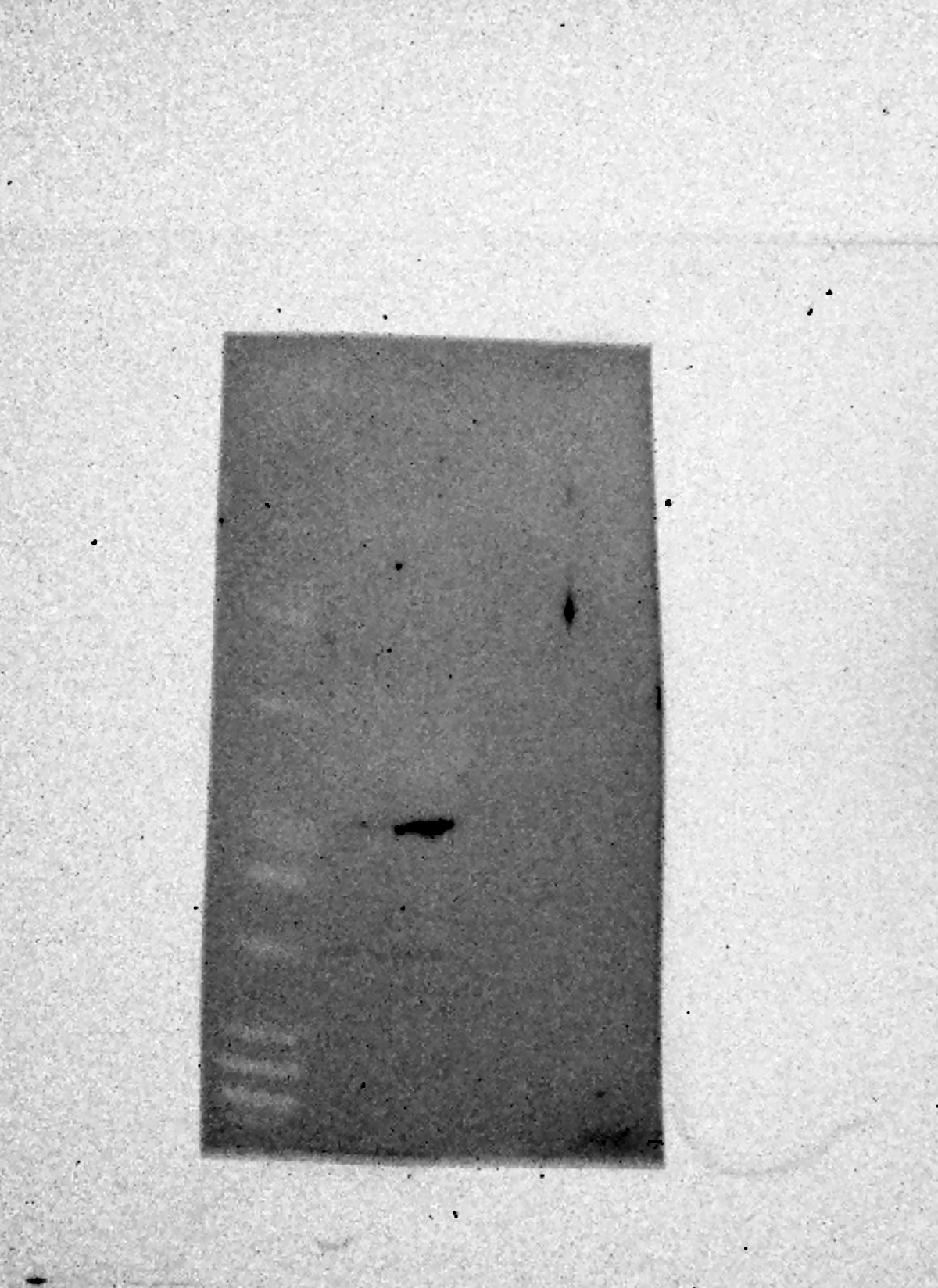

Supplement: Figure 2—figure supplement 4—source data 1. [file elife-80395-fig2-figsupp4-data1.zip › Figure 2 - figure supplement 4 - source data 1.tif]

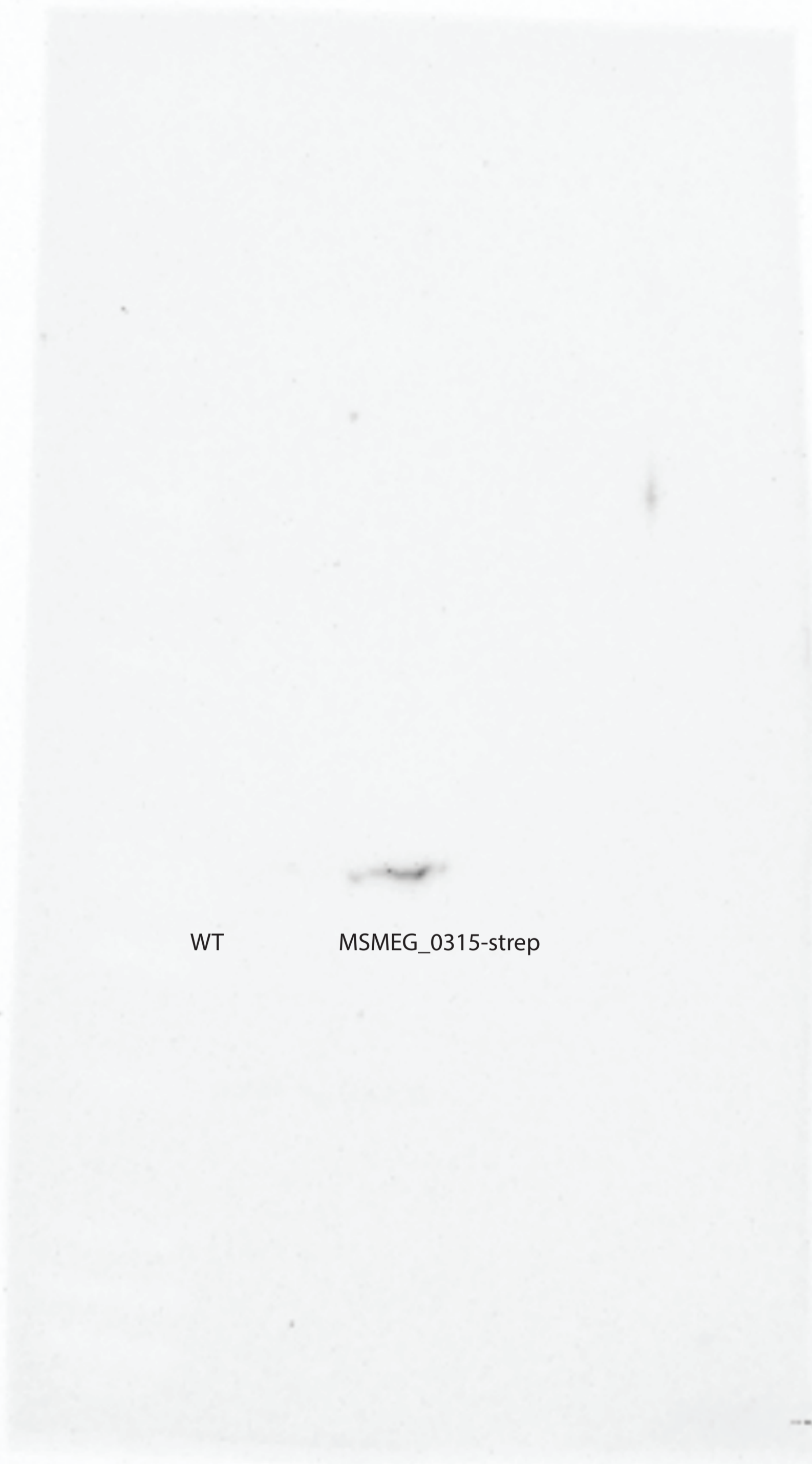

WT

MSMEG\_0315-strep

Supplement: Figure 2—figure supplement 4—source data 2. [file elife-80395-fig2-figsupp4-data2.zip › Figure 2 - figure supplement 4 - source data 2.pdf]

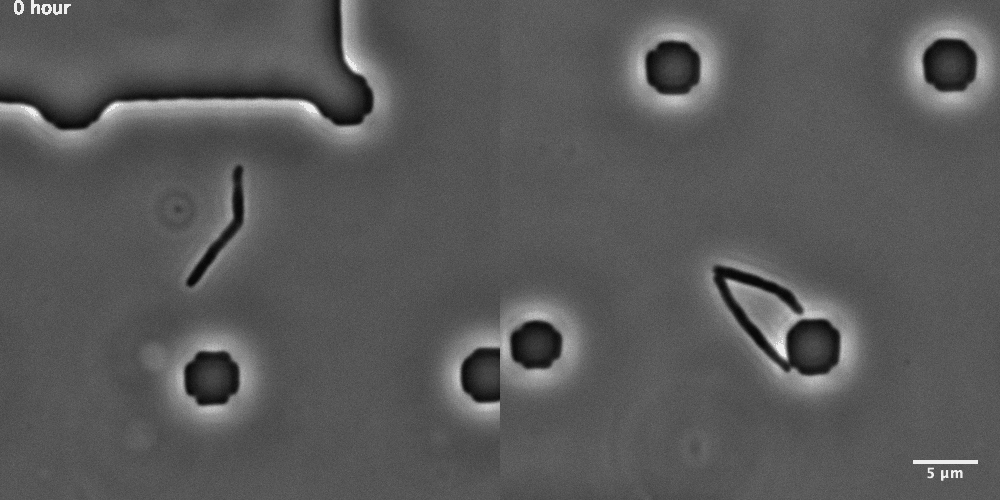

Supplement: Supplementary file 9 [file elife-80395-fig2-video1.gif]

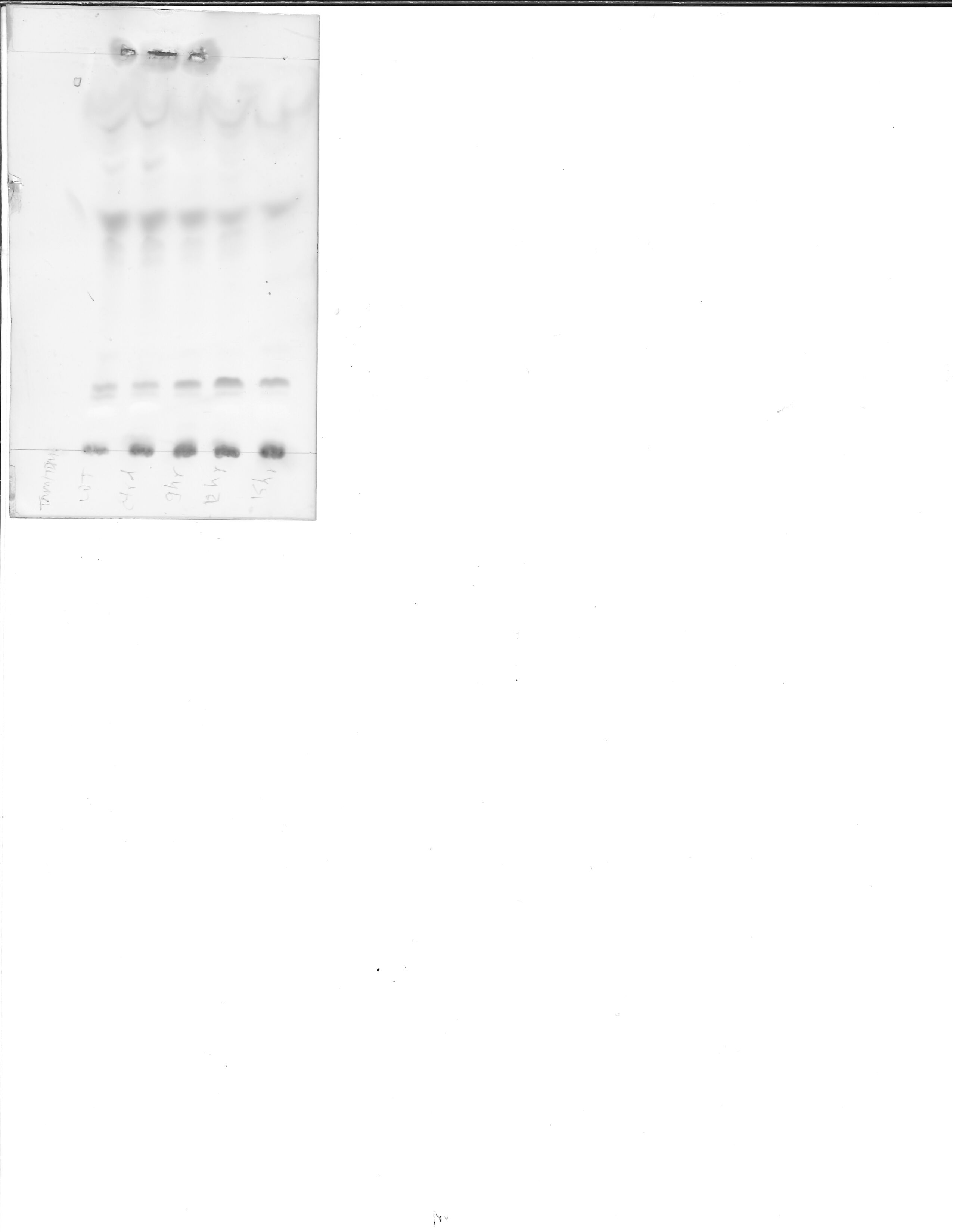

Supplement: Figure 3—source data 1. [file elife-80395-fig3-data1.zip › Figure 3 - source data 1.jpeg]

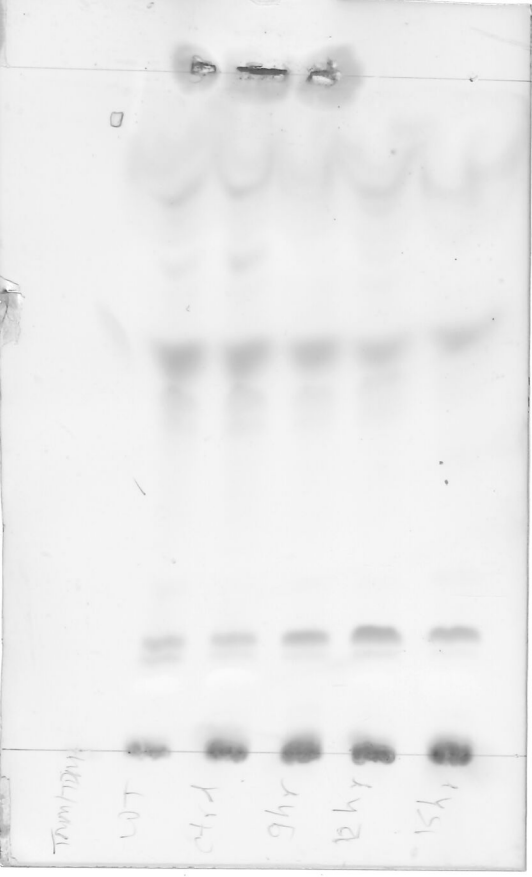

← TDM

← TMM

Supplement: Figure 3—source data 2. [file elife-80395-fig3-data2.zip › Figure 3 - source data 2.pdf]

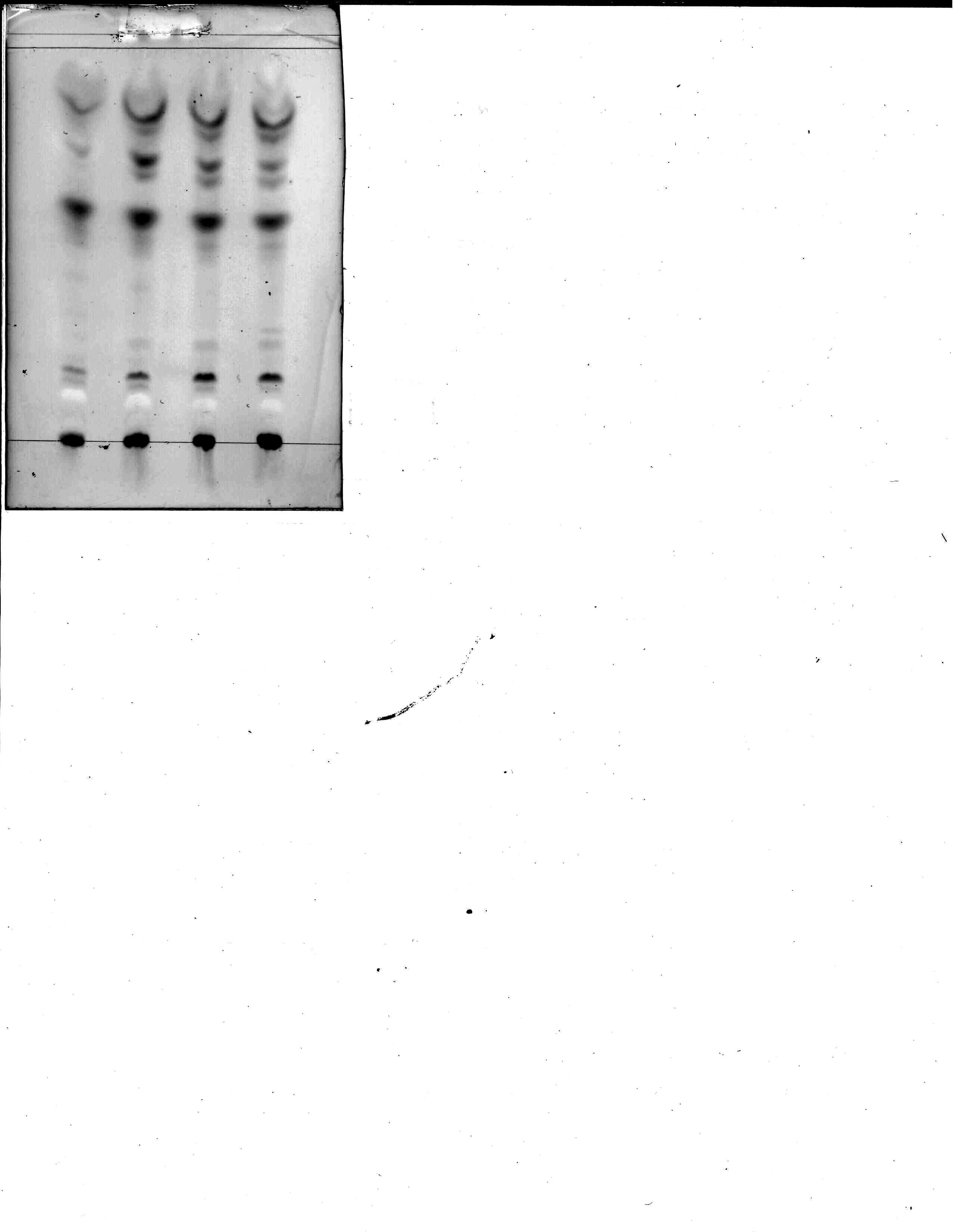

Supplement: Figure 3—source data 3. [file elife-80395-fig3-data3.zip › Figure 3 - source data 3.jpg]

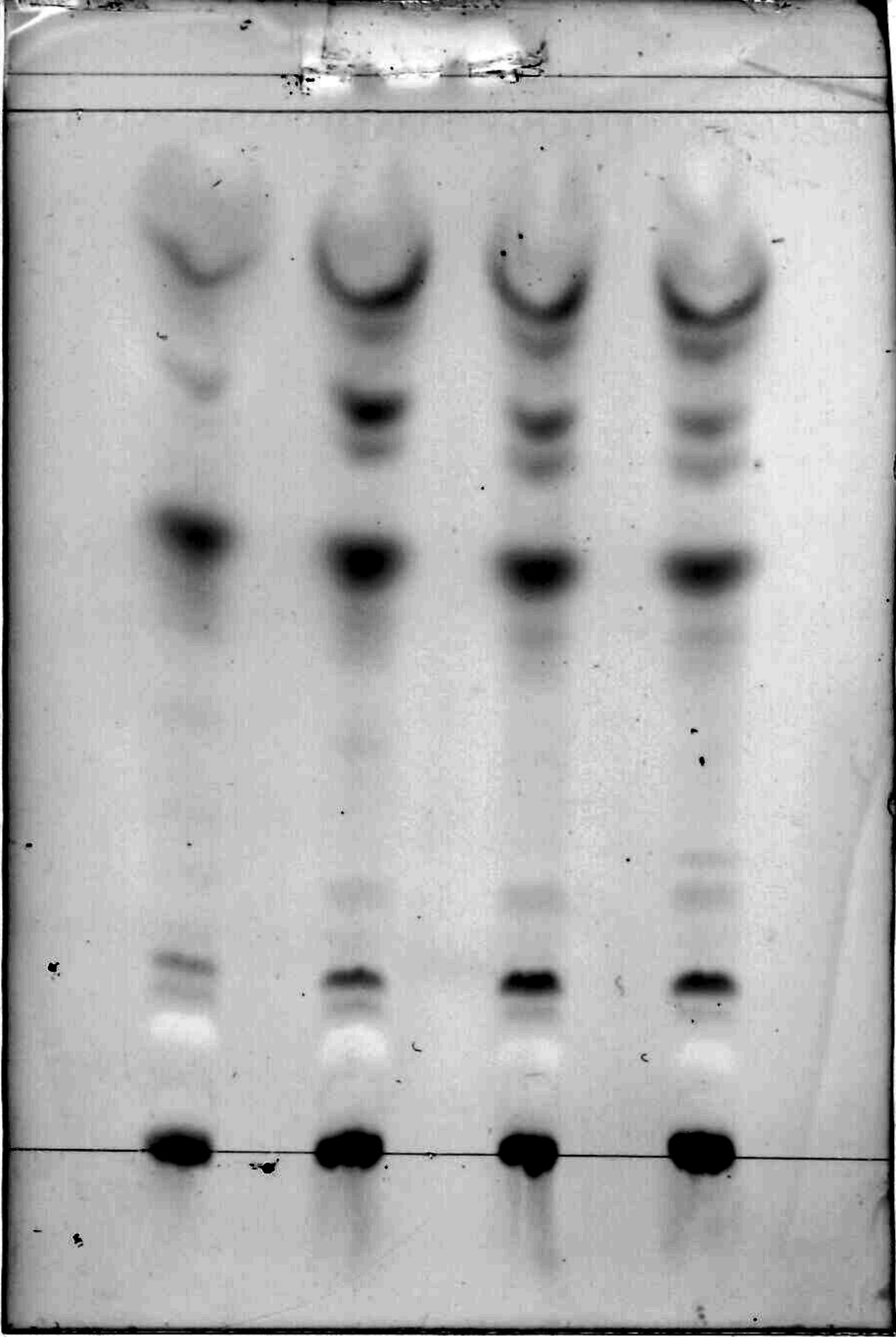

← TDM

← TMM

Supplement: Figure 3—source data 4. [file elife-80395-fig3-data4.zip › Figure 3 - source data 4.pdf]

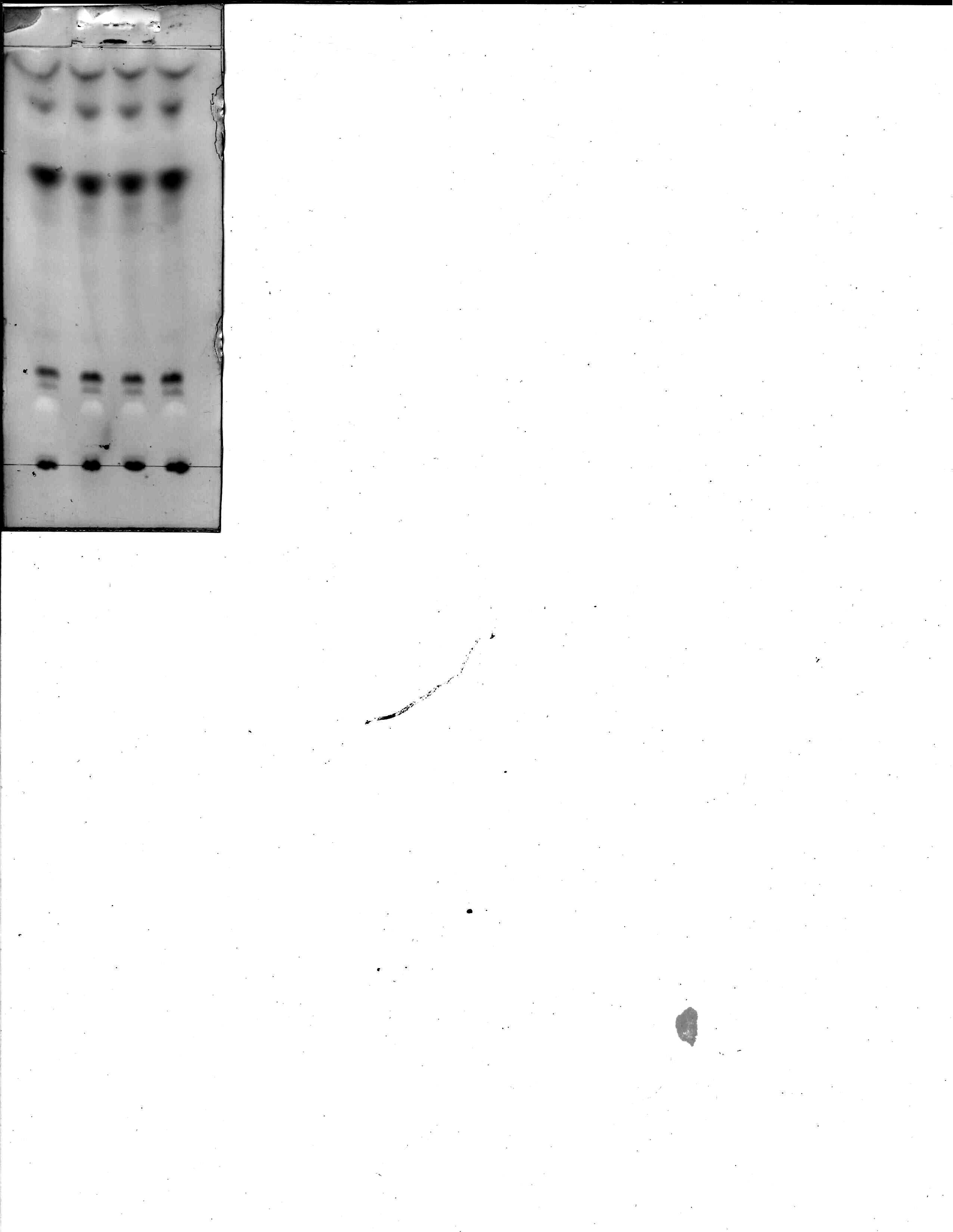

Supplement: Figure 3—source data 5. [file elife-80395-fig3-data5.zip › Figure 3 - source data 5.jpg]

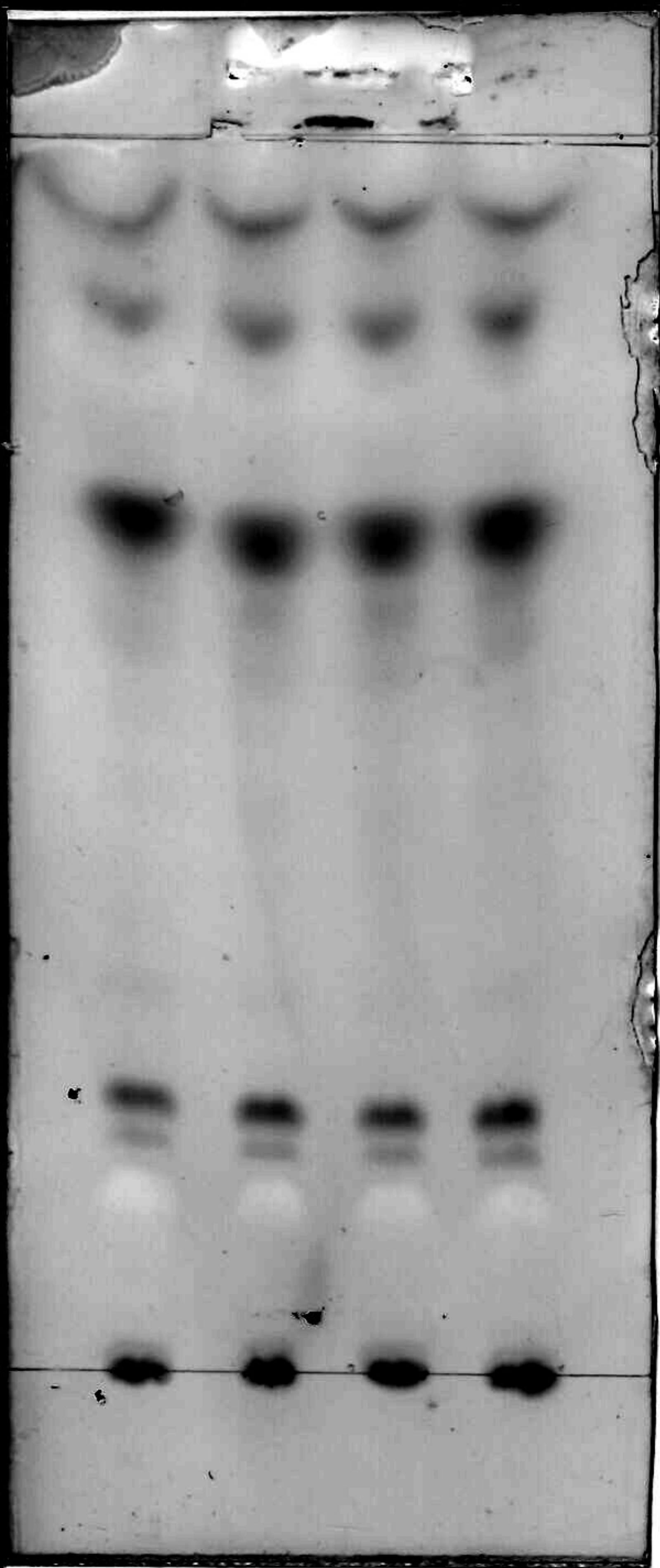

← TDM

← TMM

Supplement: Figure 3—source data 6. [file elife-80395-fig3-data6.zip › Figure 3 - source data 6.pdf]

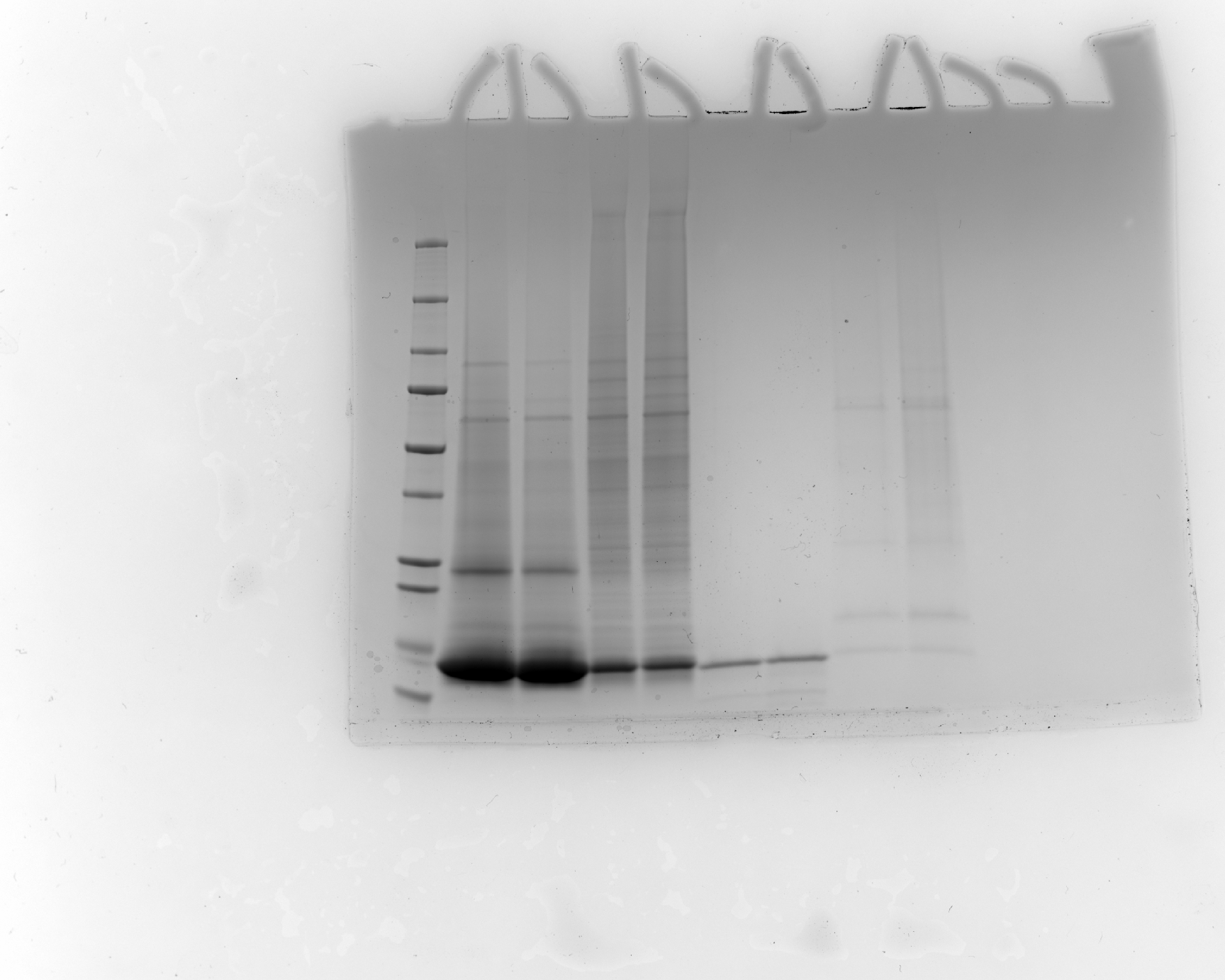

Supplement: Figure 3—figure supplement 1—source data 1. [file elife-80395-fig3-figsupp1-data1.zip › Figure 3 - figure supplement 1 - source data 1.tif]

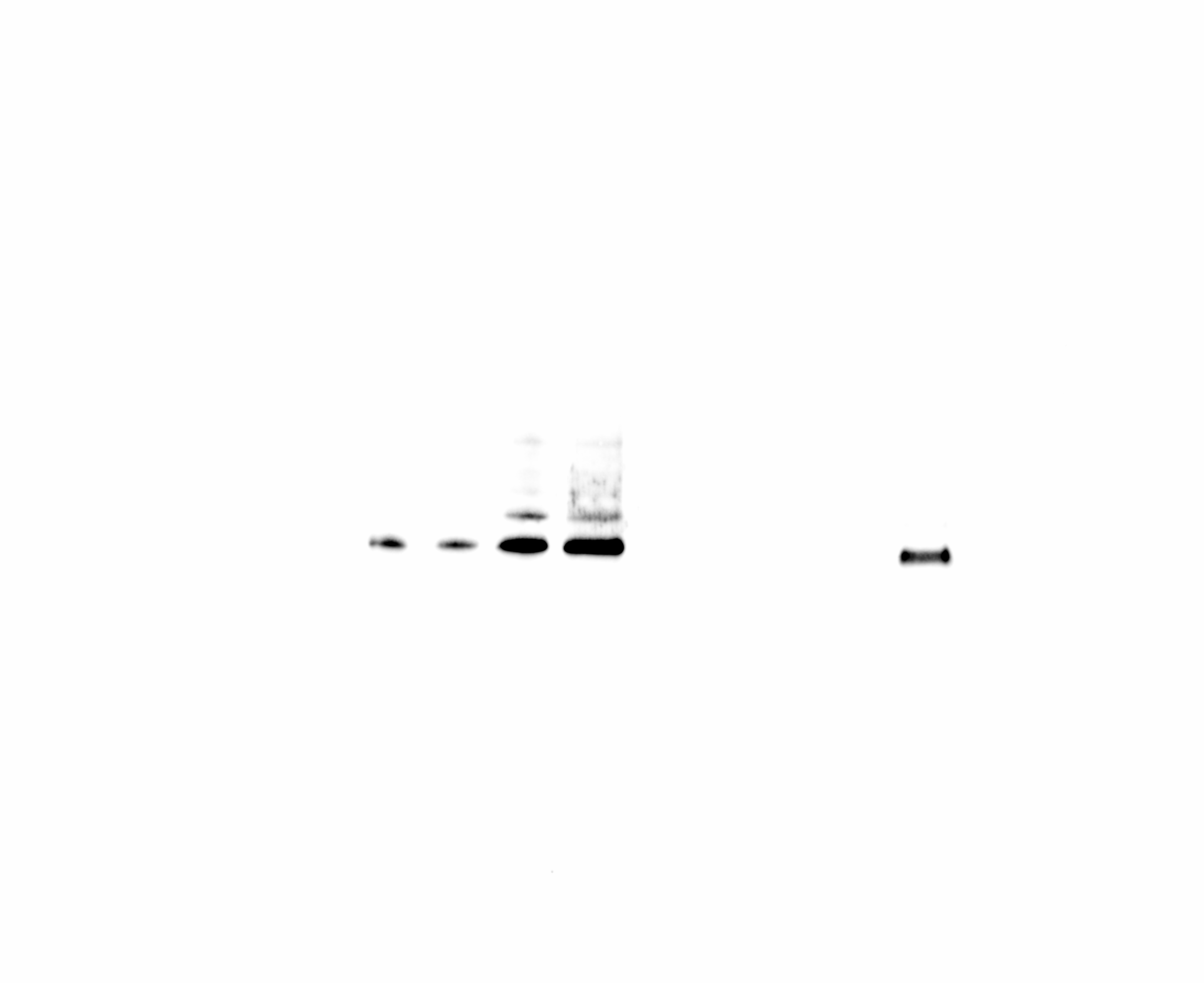

Supplement: Figure 3—figure supplement 1—source data 2. [file elife-80395-fig3-figsupp1-data2.zip › Figure 3 - figure supplement 1 - source data 2.tif]

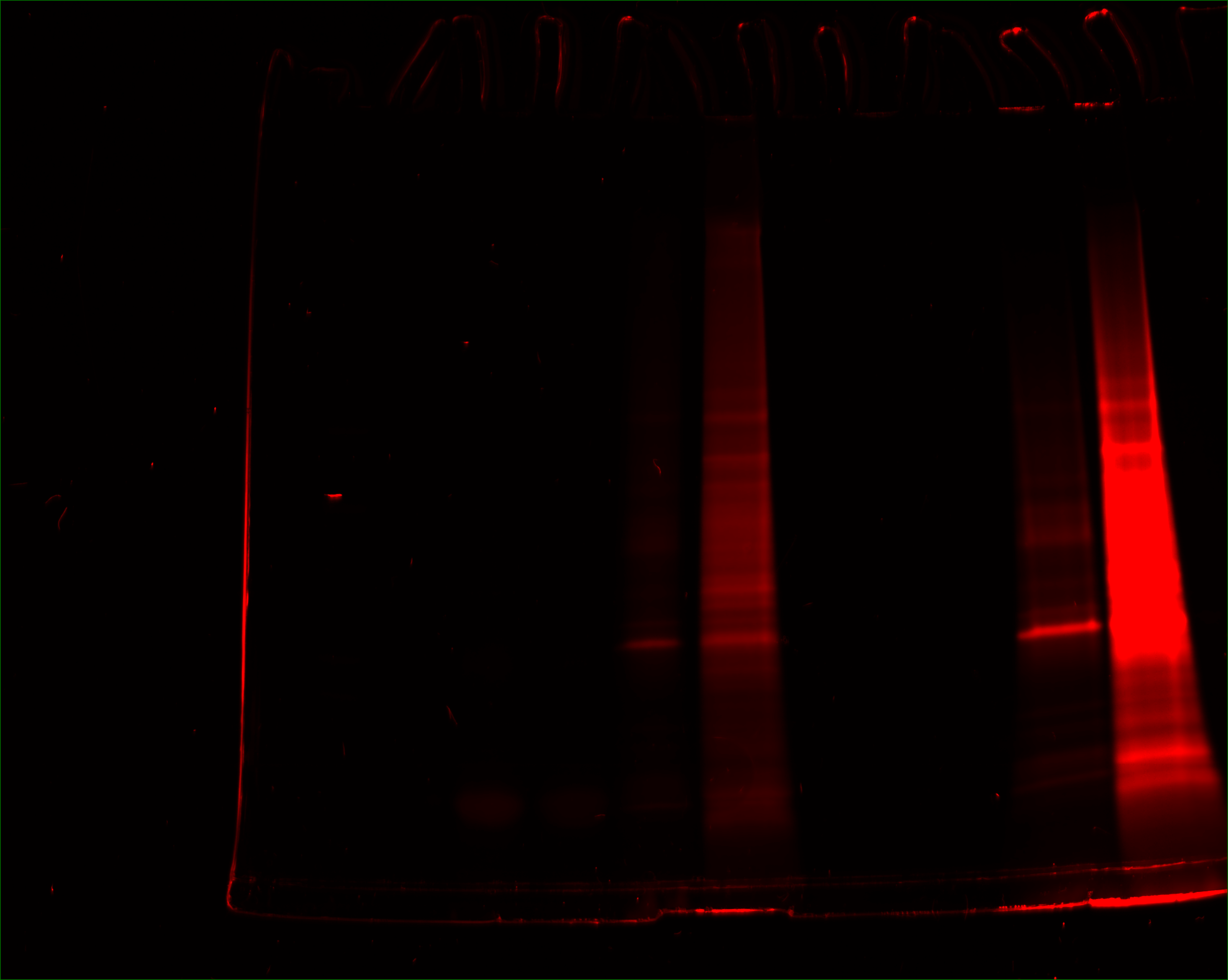

Supplement: Figure 3—figure supplement 1—source data 3. [file elife-80395-fig3-figsupp1-data3.zip › Figure 3 - figure supplement 1 - source data 3.bmp]

10

8

+

+

+

MSMEG\_0317

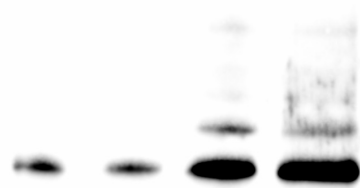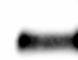

kDa  
250  
150  
100  
75  
50  
37  
25  
20  
15  
10

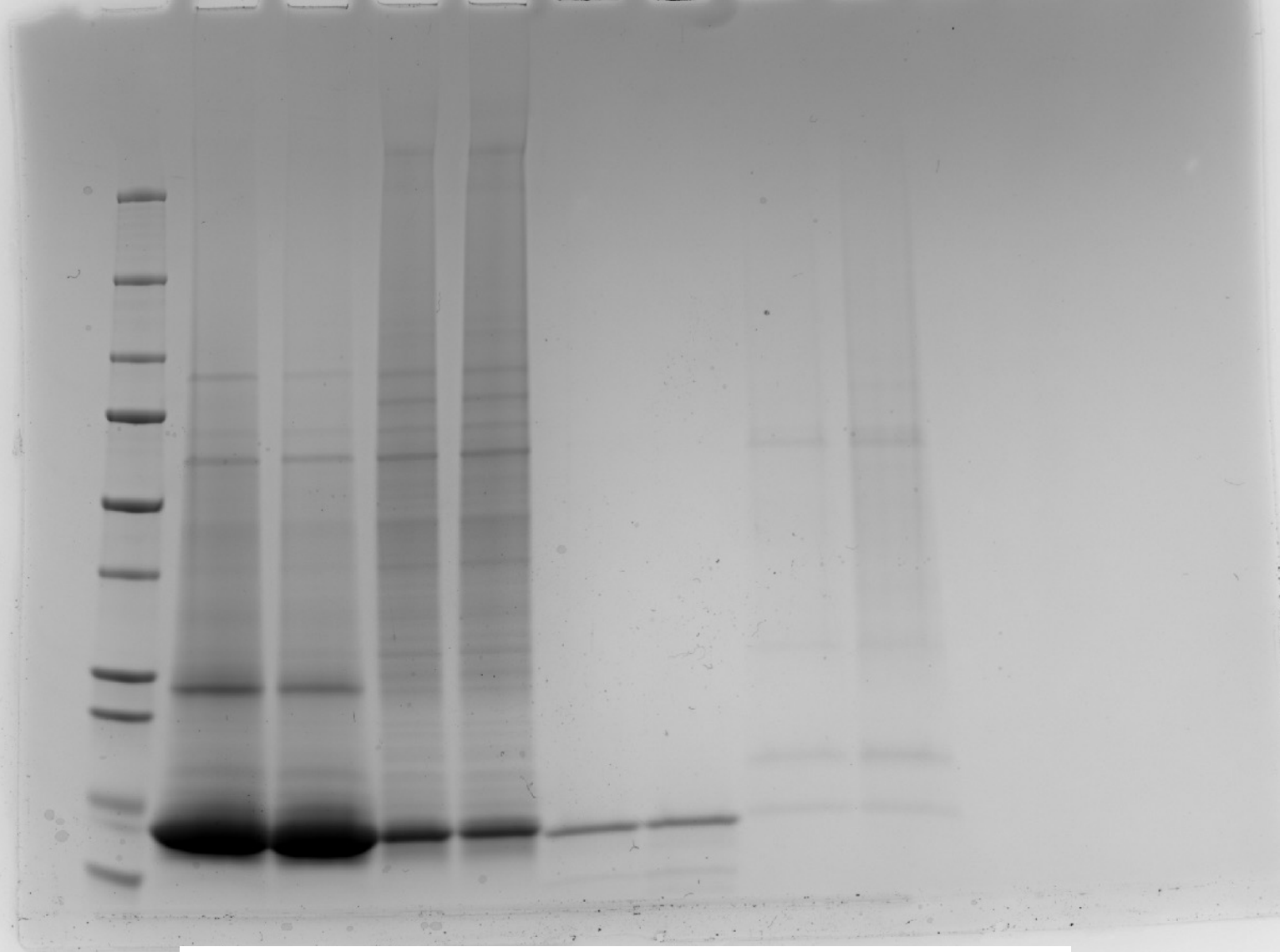

Lane

N-x-AlkTMM-C15

UV (365nm)

CuAAC (AzTB)

| 1 | 2 | 3 | 4 | 5 | 6 | 7 | 8 |
|---|---|---|---|---|---|---|---|
| - | - | + | + | - | - | + | + |
| - | + | - | + | - | + | - | + |
| + | + | + | + | + | + | + | + |

**kDa**  
**250**  
**150**  
**100**  
**75**  
**50**  
**37**  
**25**  
**20**  
**15**  
**10**

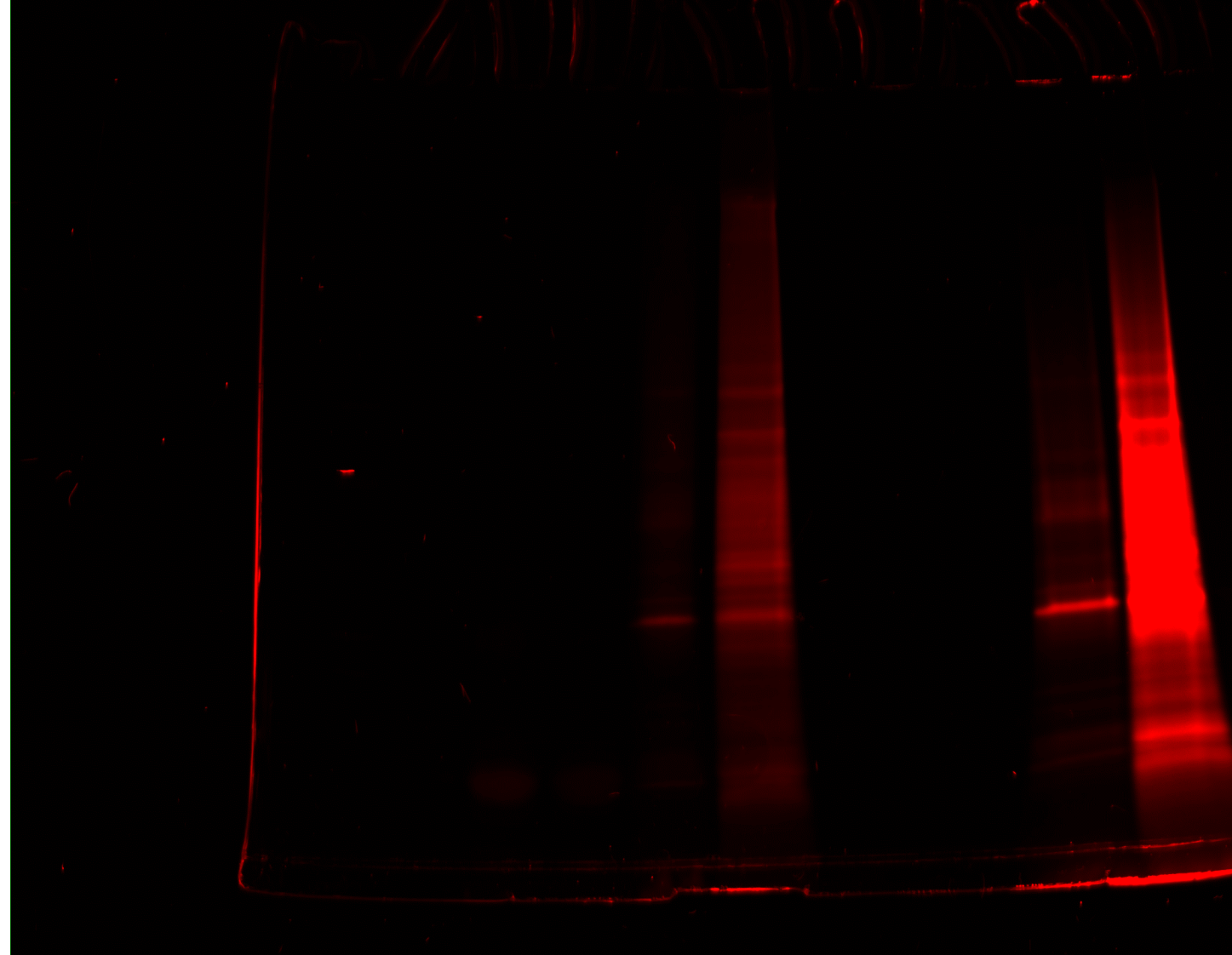

| Lane           | 1 | 2 | 3 | 4 | 5 | 6 | 7 | 8 |
|----------------|---|---|---|---|---|---|---|---|
| N-x-AlkTMM-C15 | - | - | + | + | - | - | + | + |
| UV (365nm)     | - | + | - | + | - | + | - | + |
| CuAAC (AzTB)   | + | + | + | + | + | + | + | + |

Supplement: Figure 3—figure supplement 1—source data 4. [file elife-80395-fig3-figsupp1-data4.zip › Figure 3 - figure supplement 1 - source data 4.pdf]

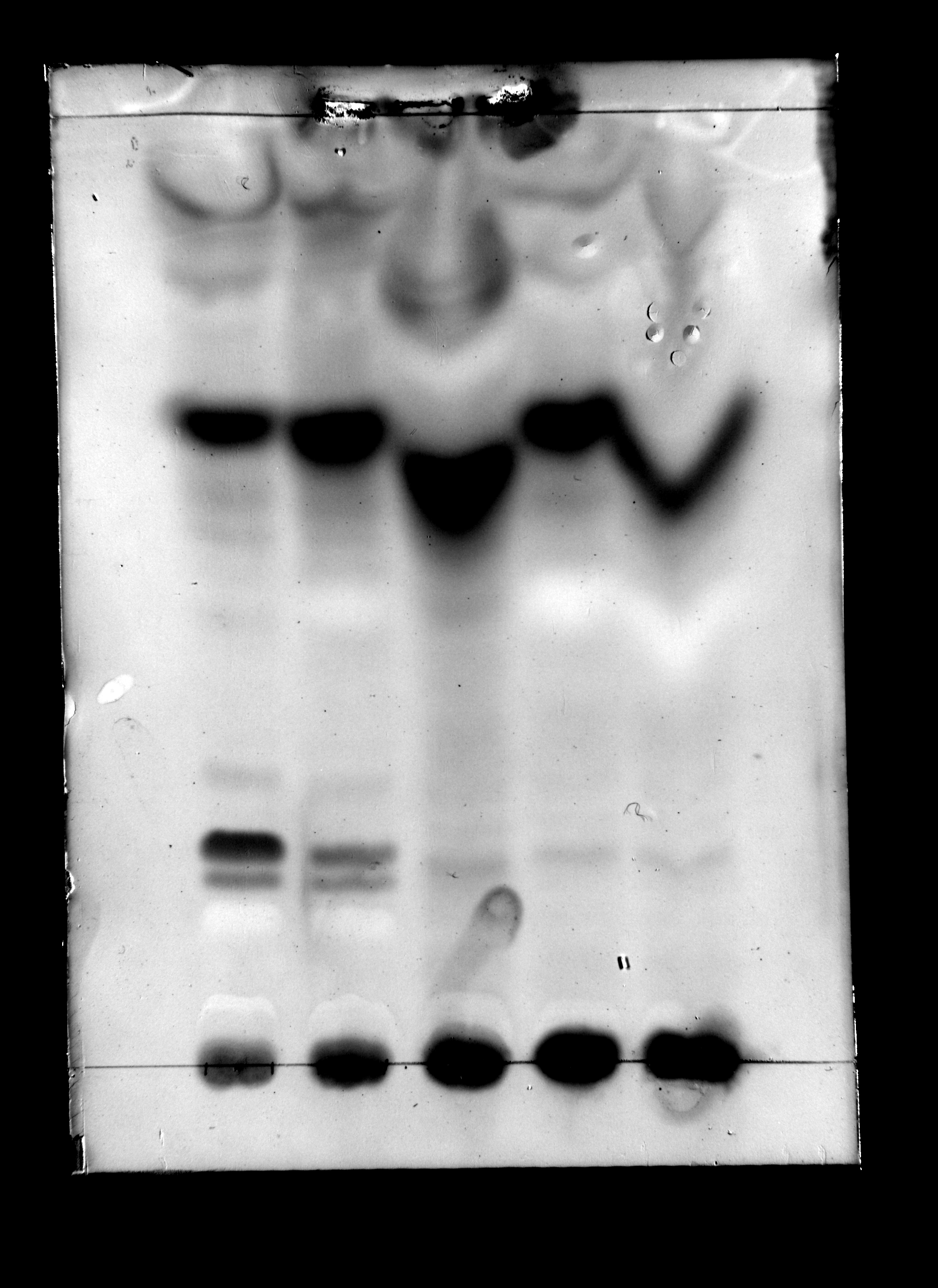

Supplement: Figure 3—figure supplement 3—source data 1. [file elife-80395-fig3-figsupp3-data1.zip › Figure 3 - figure supplement 3 - source data 1.tif]

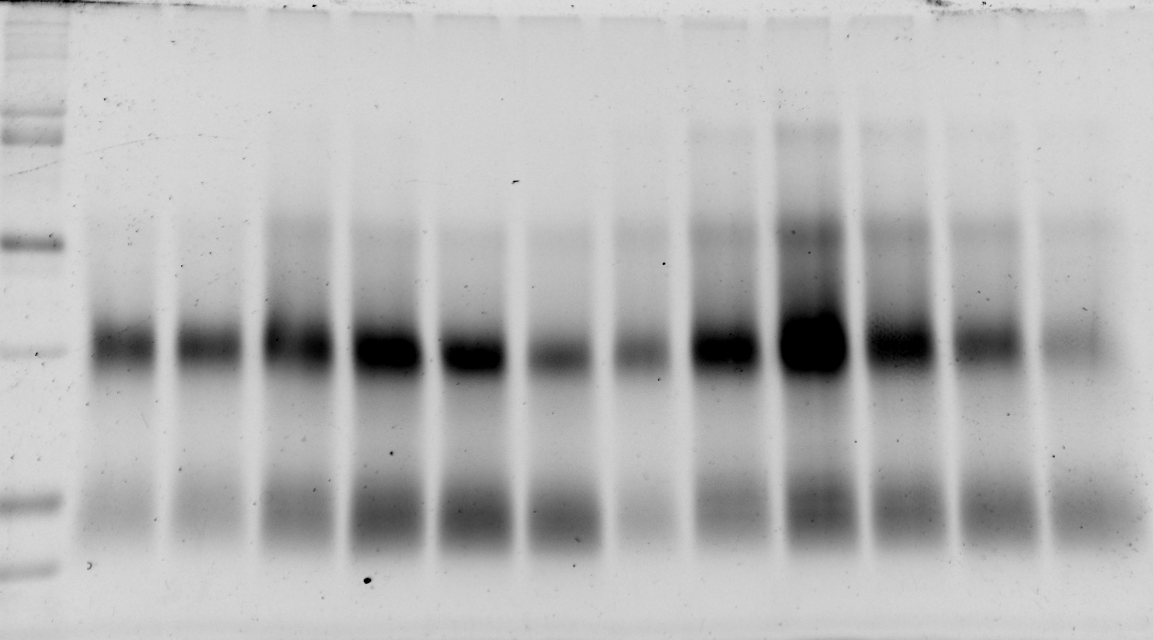

Supplement: Figure 4—source data 1. [file elife-80395-fig4-data1.zip › Figure 4 - source data 1.tif]

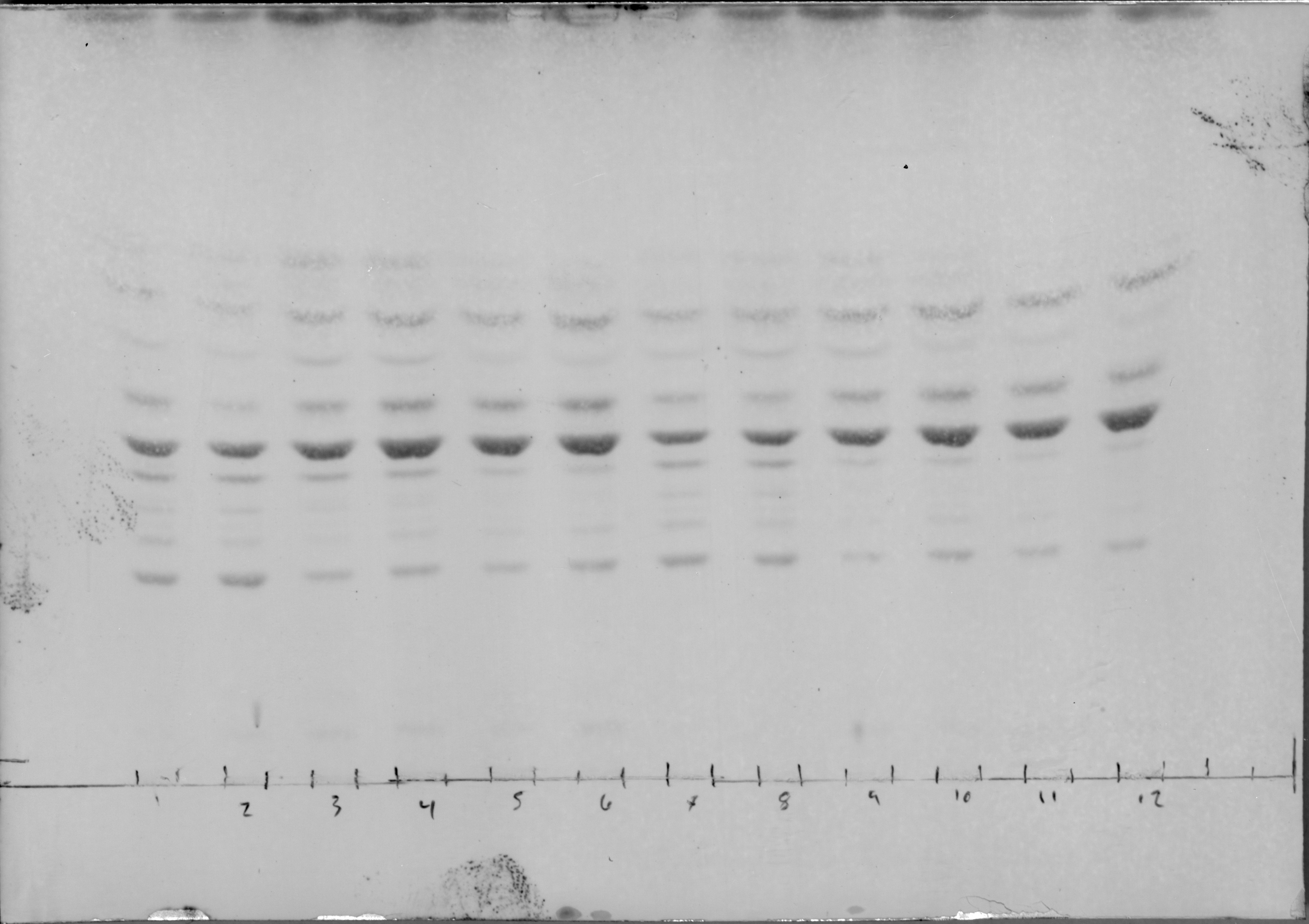

Supplement: Figure 4—source data 2. [file elife-80395-fig4-data2.zip › Figure 4 - source data 2.tif]

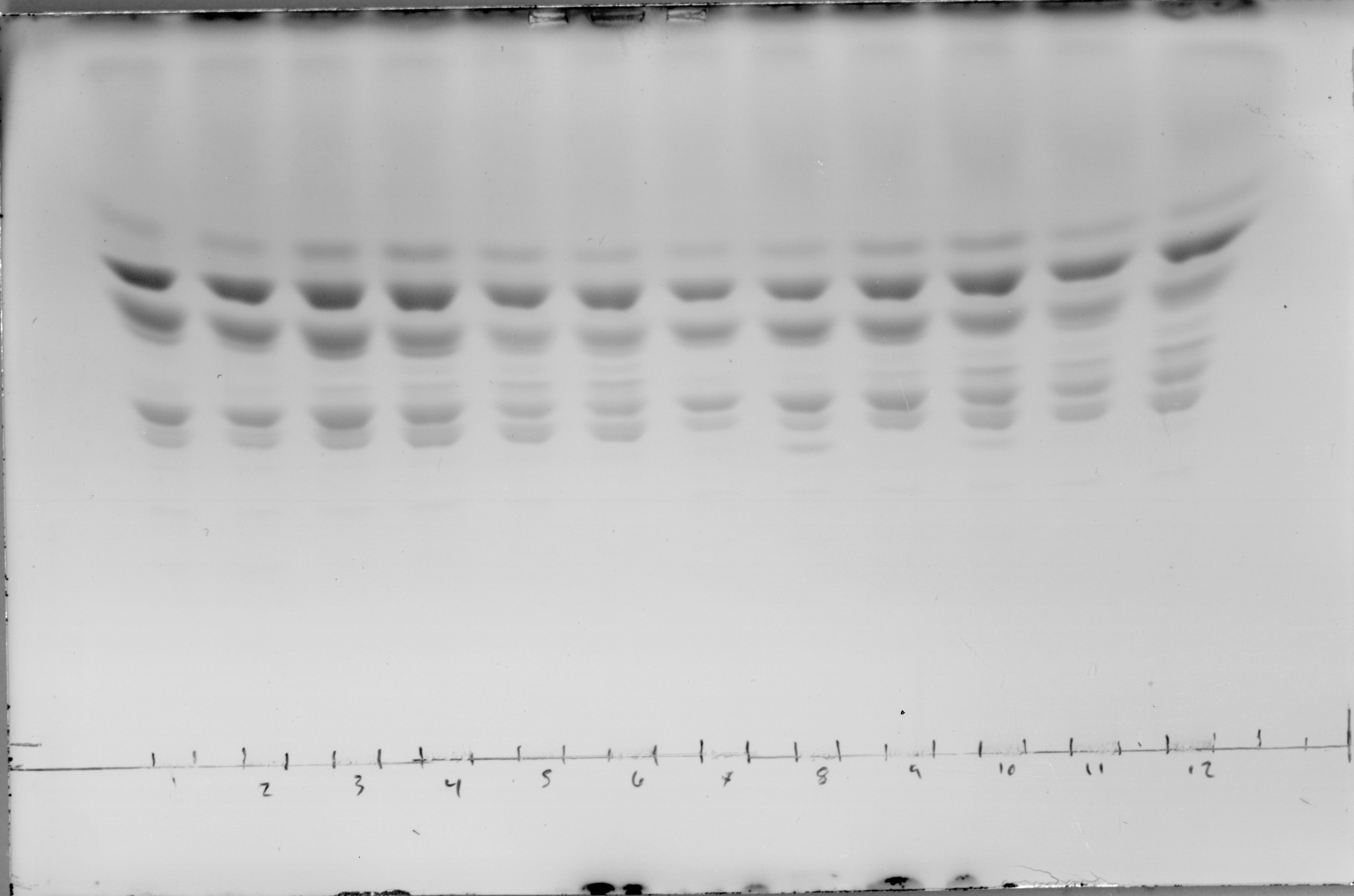

Supplement: Figure 4—source data 3. [file elife-80395-fig4-data3.zip › Figure 4 - source data 3.tif]

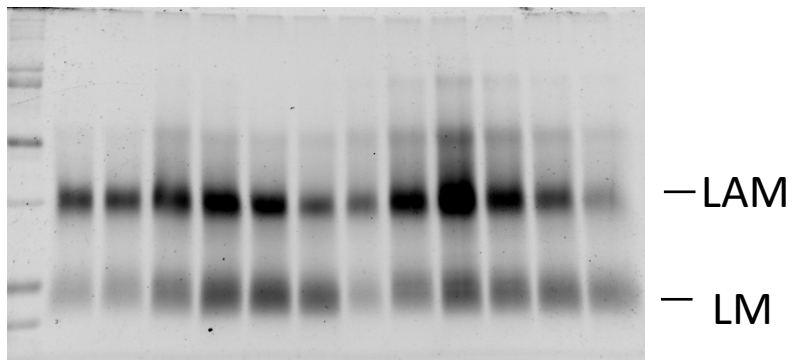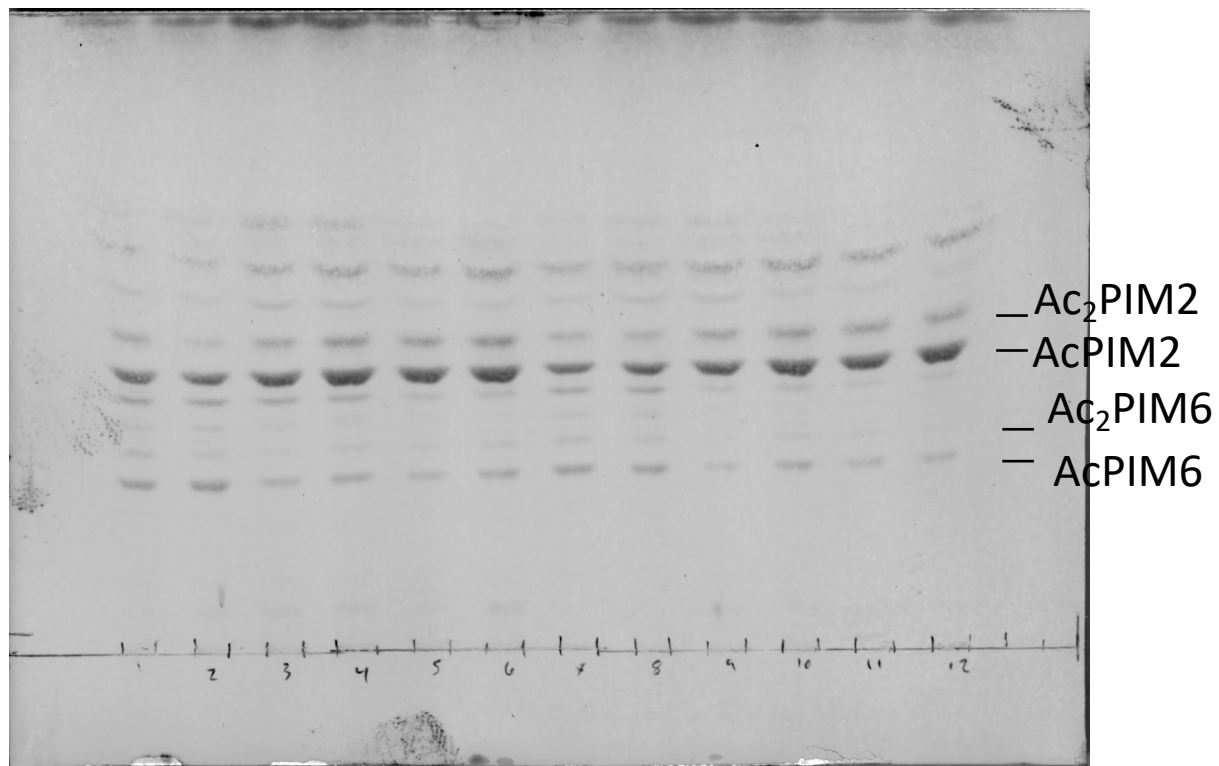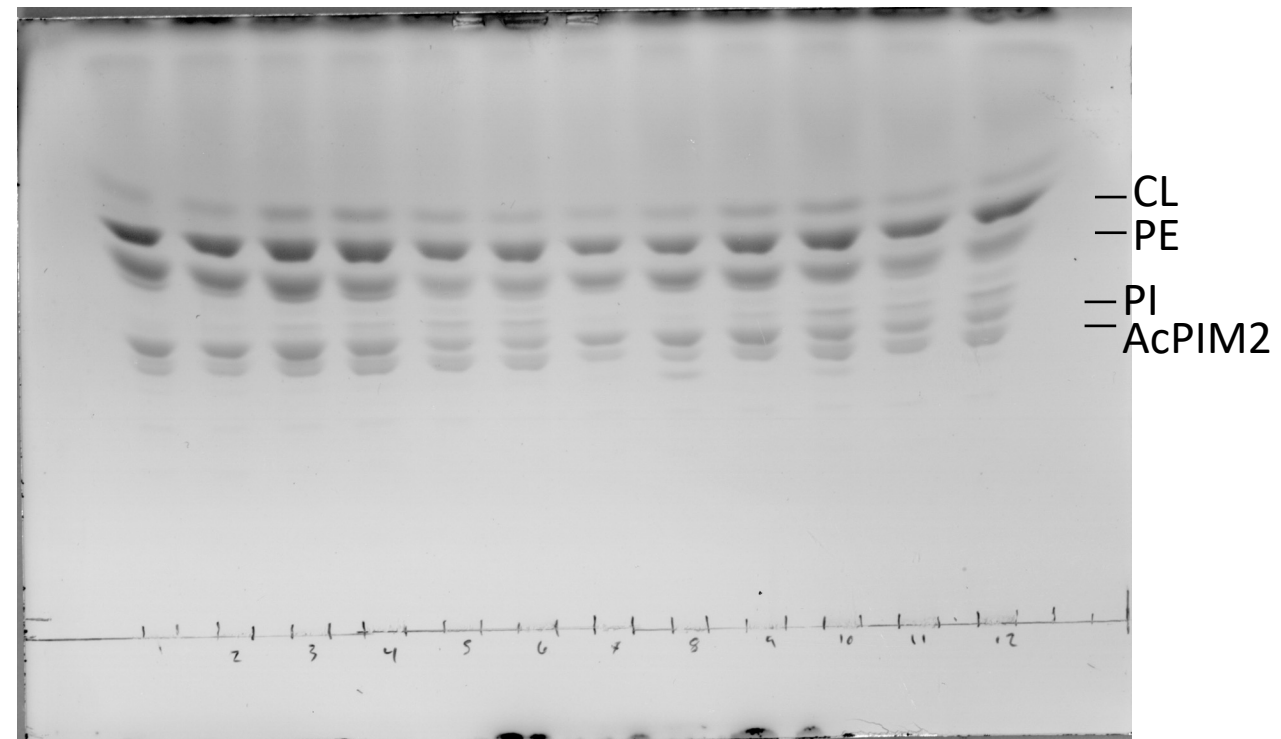

Supplement: Figure 4—source data 4. [file elife-80395-fig4-data4.zip › Figure 4 - source data 4.pdf]

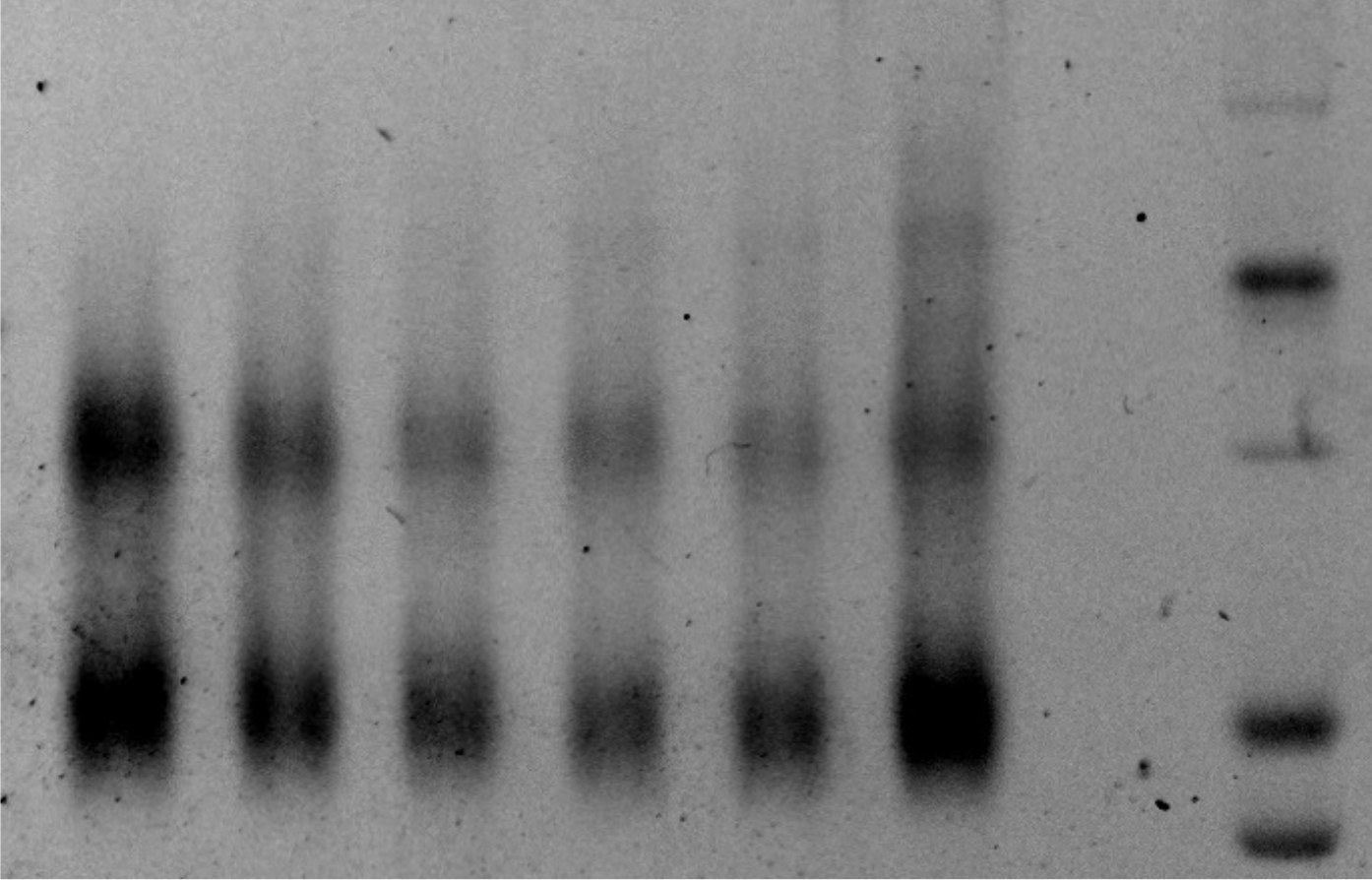

Supplement: Figure 4—source data 6. [file elife-80395-fig4-data6.zip › Figure 4 - source data 6.tif]

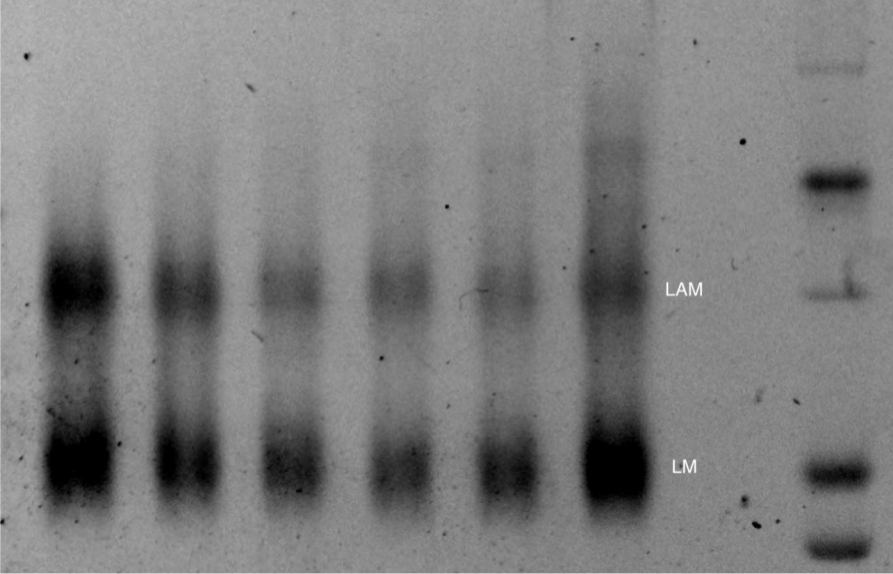

Supplement: Figure 4—source data 7. [file elife-80395-fig4-data7.zip › Figure 4 - source data 7.pdf]

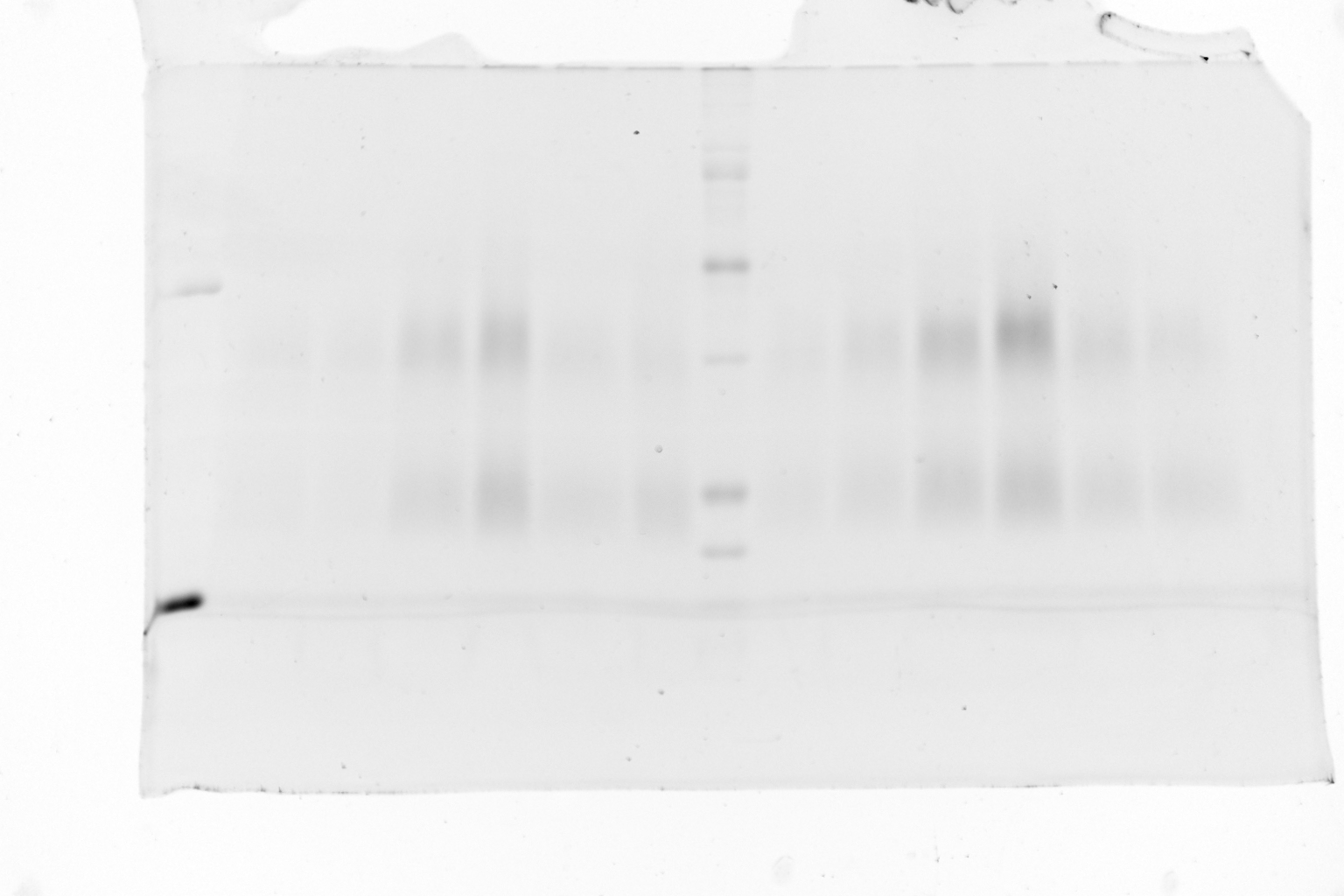

Supplement: Figure 4—figure supplement 1—source data 1. [file elife-80395-fig4-figsupp1-data1.zip › Figure 4 - figure supplement 1 - source data 1.tif]

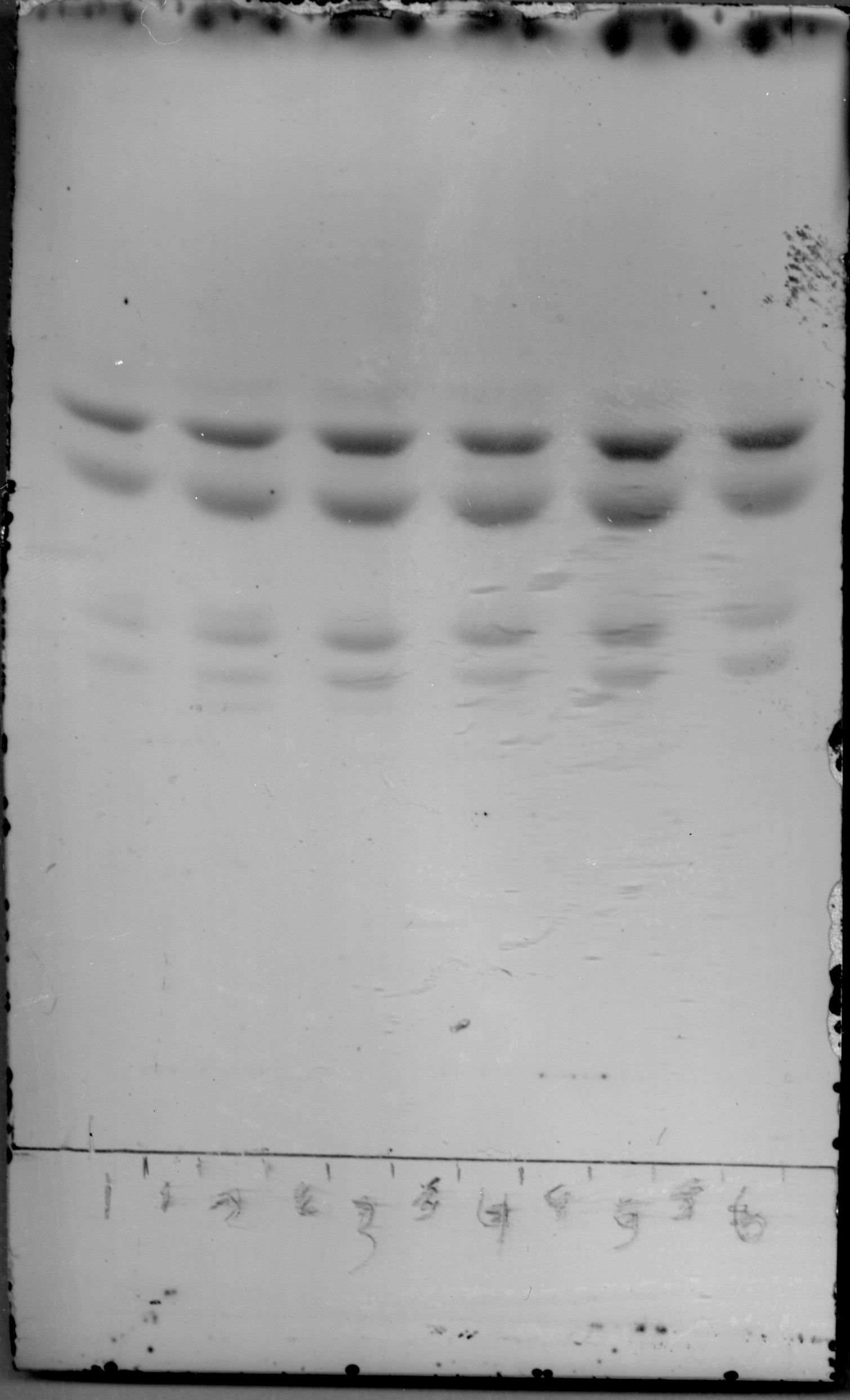

Supplement: Figure 4—figure supplement 1—source data 2. [file elife-80395-fig4-figsupp1-data2.zip › Figure 4 - figure supplement 1 - source data 2.tif]

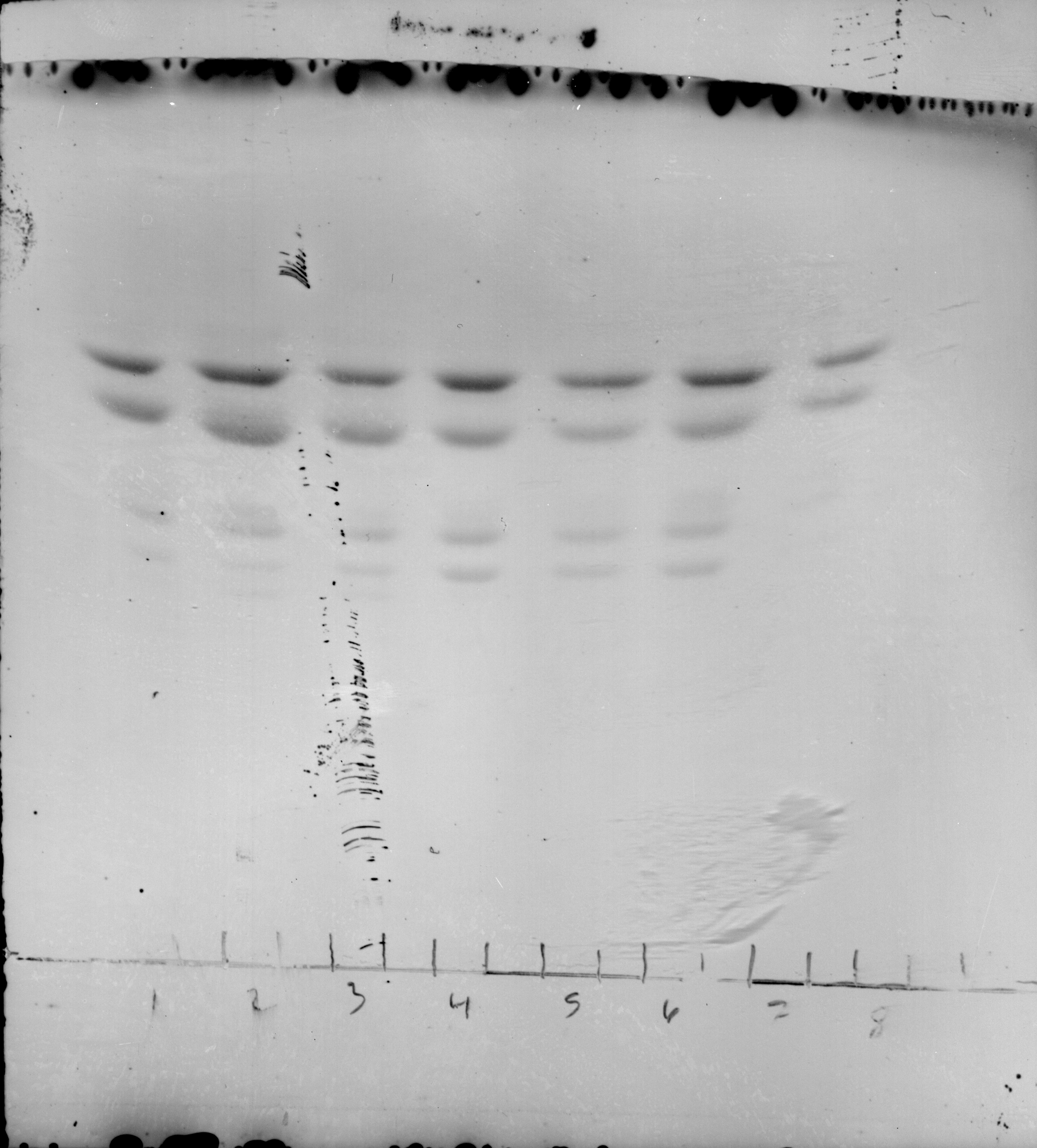

Supplement: Figure 4—figure supplement 1—source data 3. [file elife-80395-fig4-figsupp1-data3.zip › Figure 4 - figure supplement 1 - source data 3.tif]

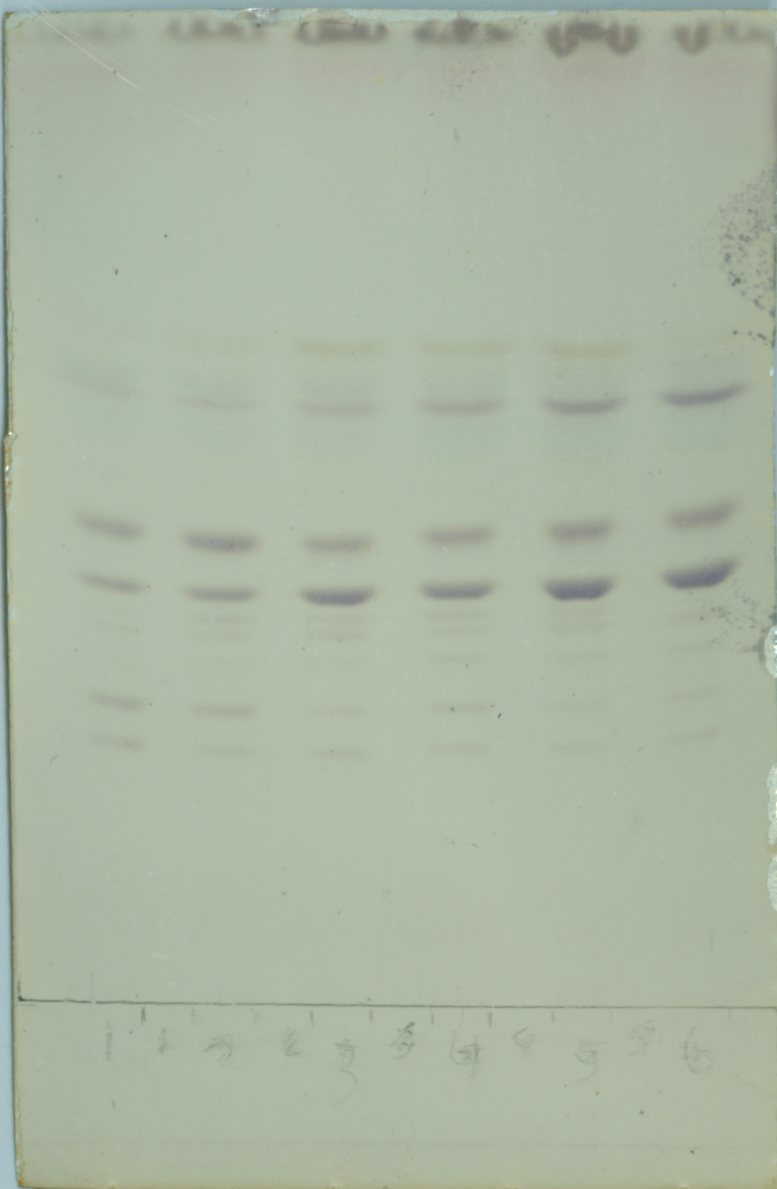

Supplement: Figure 4—figure supplement 1—source data 4. [file elife-80395-fig4-figsupp1-data4.zip › Figure 4 - figure supplement 1 - source data 4.tiff]

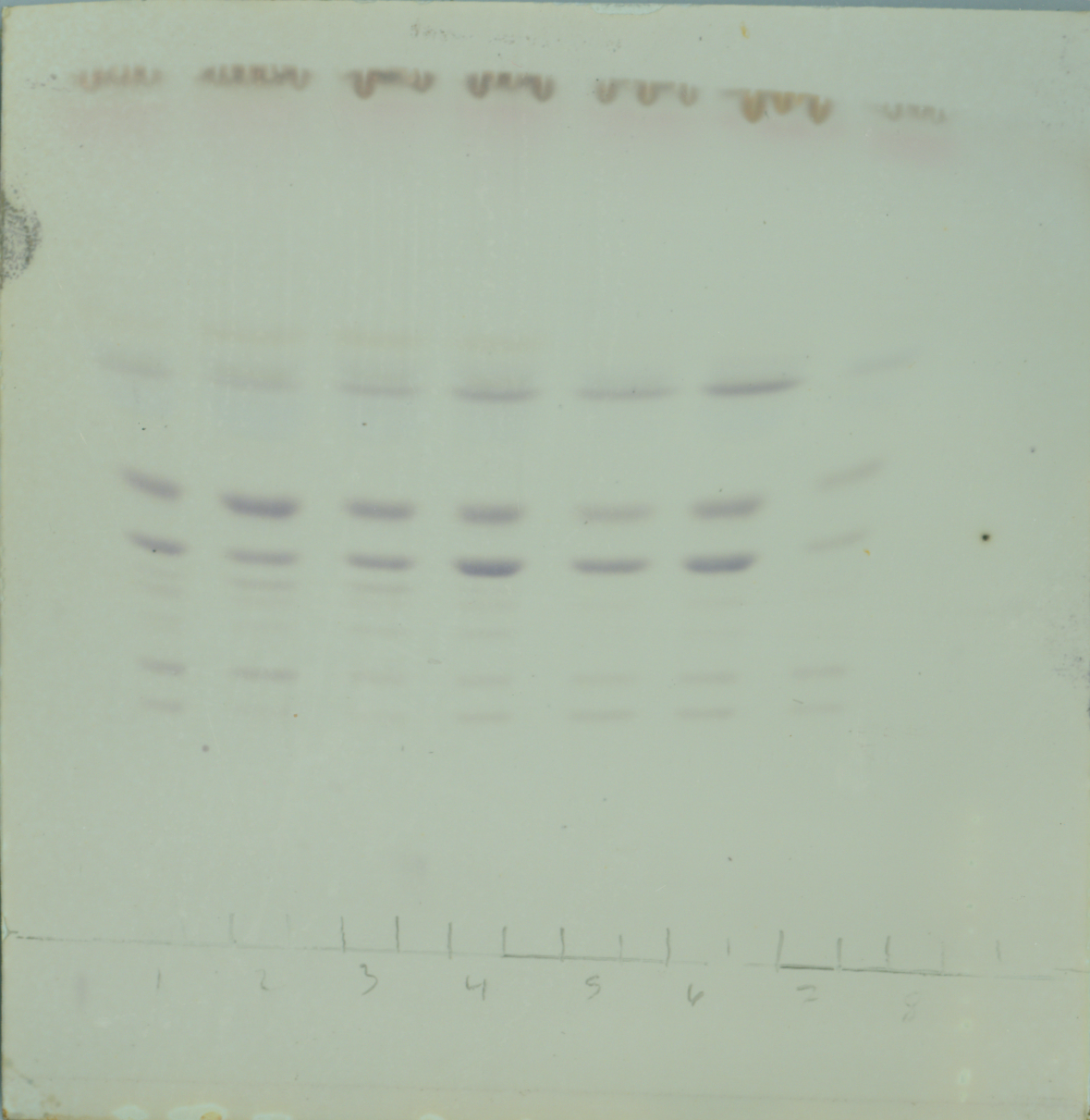

Supplement: Figure 4—figure supplement 1—source data 5. [file elife-80395-fig4-figsupp1-data5.zip › Figure 4 - figure supplement 1 - source data 5.tiff]

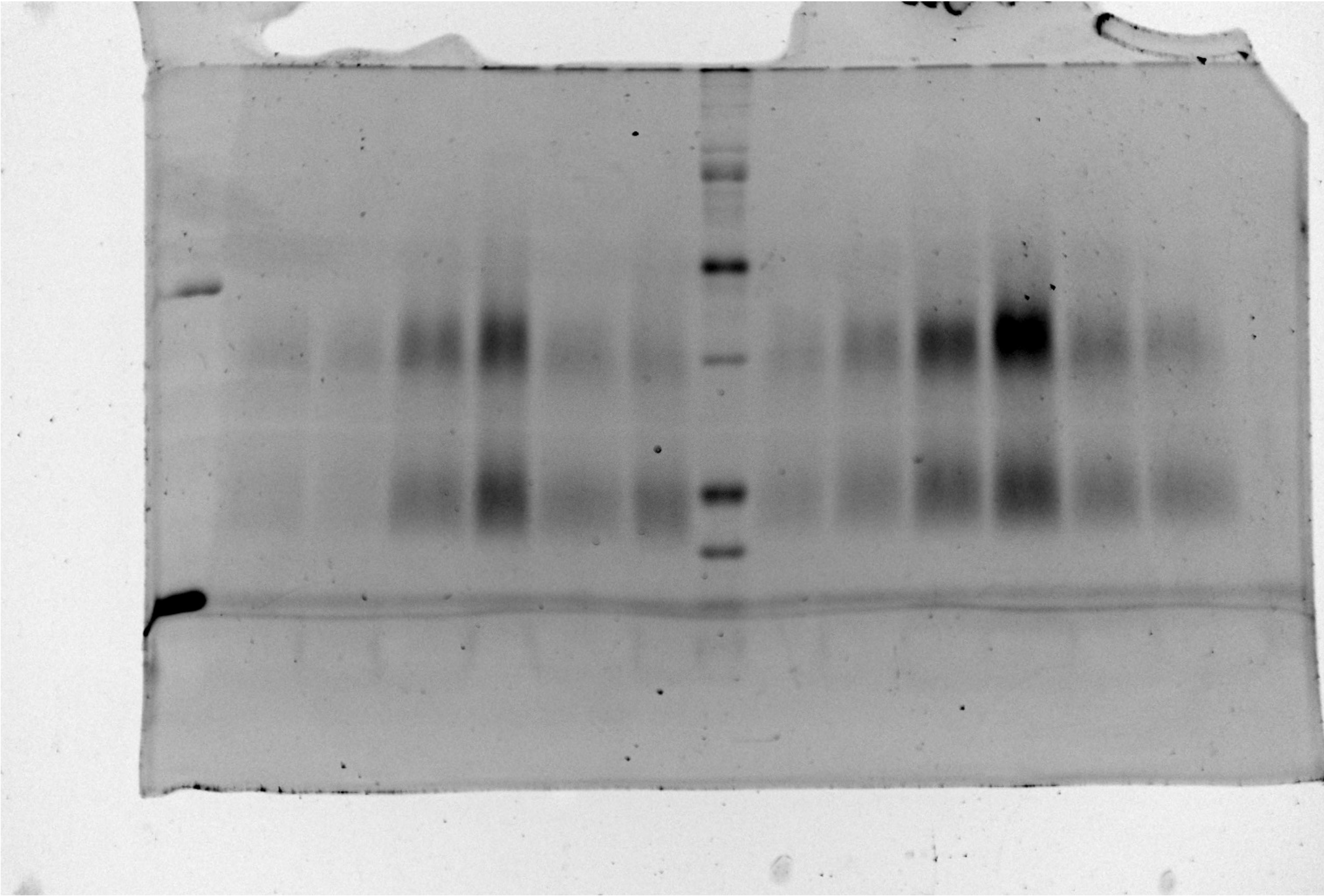

— LAM

— LM

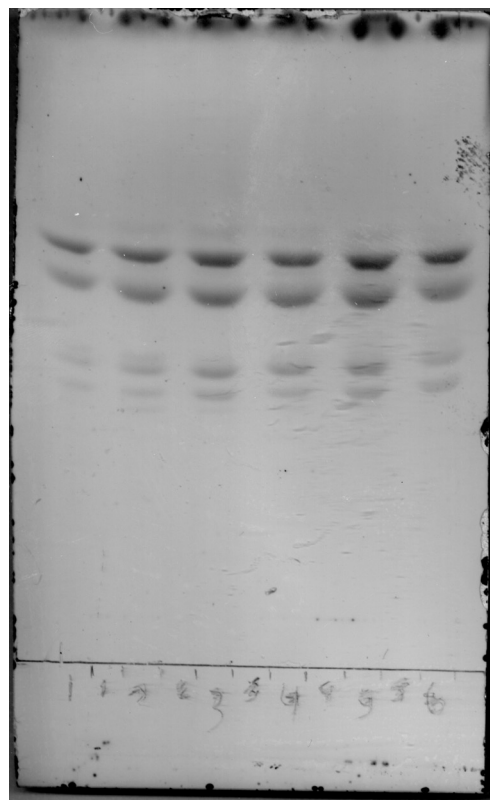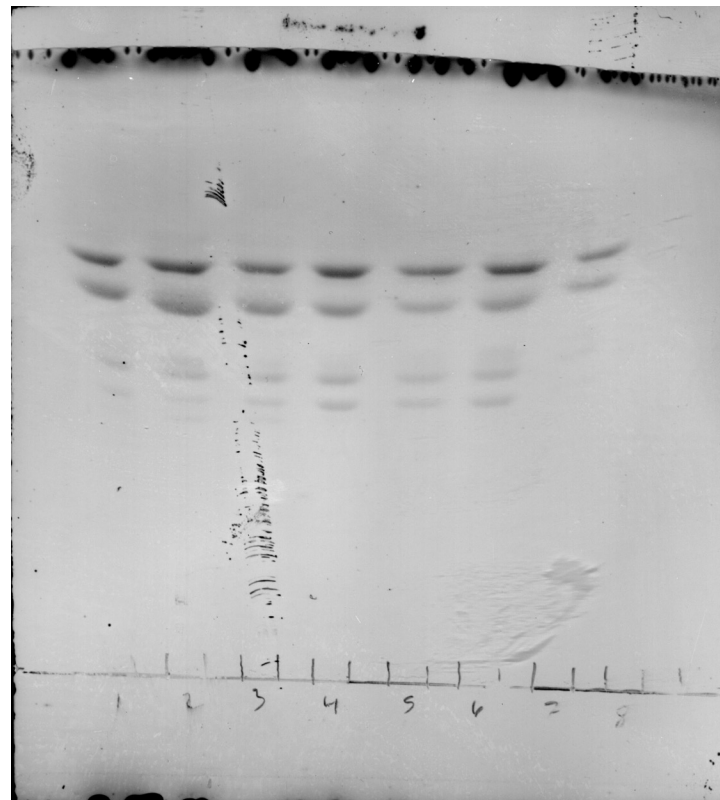

—CL  
—PE  
  
—PI  
—AcPIM2

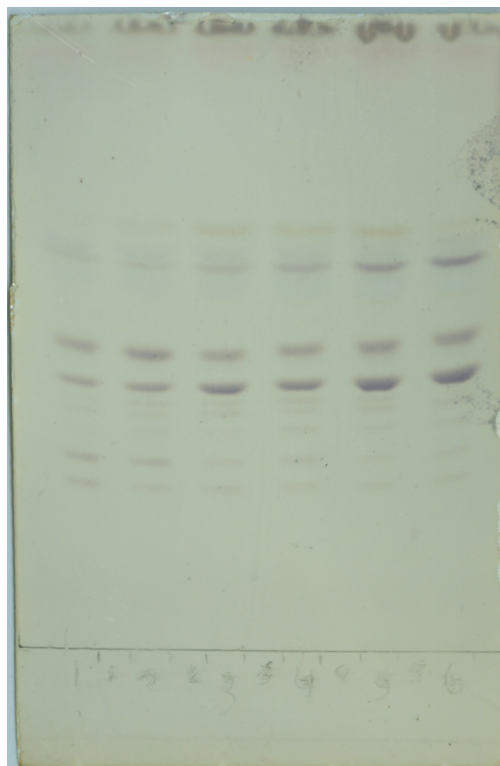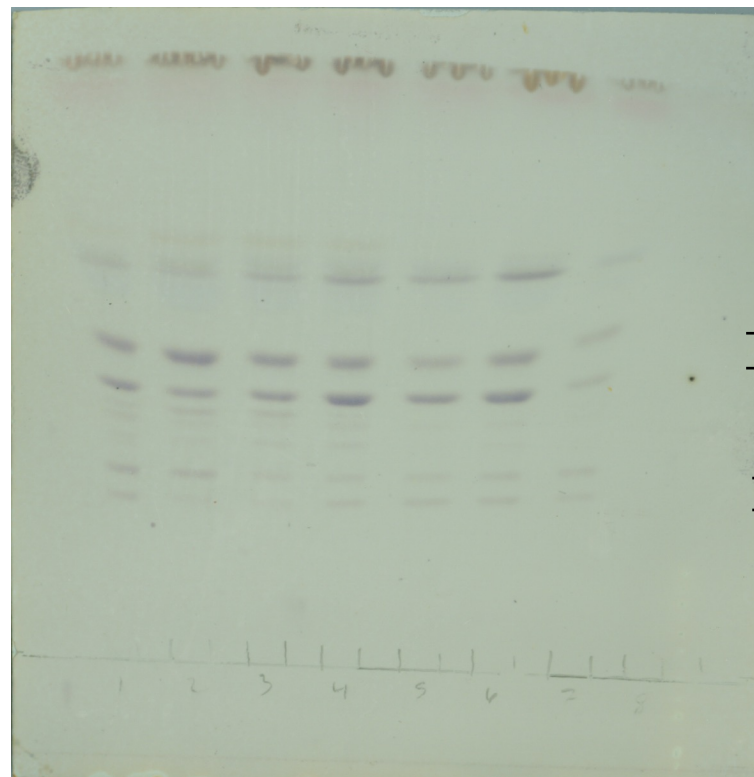

— Ac<sub>2</sub>PIM2  
— AcPIM2  
—  
— AcPIM6

Supplement: Figure 4—figure supplement 1—source data 6. [file elife-80395-fig4-figsupp1-data6.zip › Figure 4 - figure supplement 1 - source data 6.pdf]
