# Supplementary material for: An essential periplasmic protein coordinates lipid trafficking and is required for asymmetric polar growth in mycobacteria
Source: eLife. 2022 Nov 8;11:e80395. doi: 10.7554/eLife.80395 (PMC9678360; doi:10.7554/eLife.80395)
Supplement: Figure 2—figure supplement 2—source data 2. [file elife-80395-fig2-figsupp2-data2.zip › Figure 2 - figure supplement 2 - source data 2.pdf]

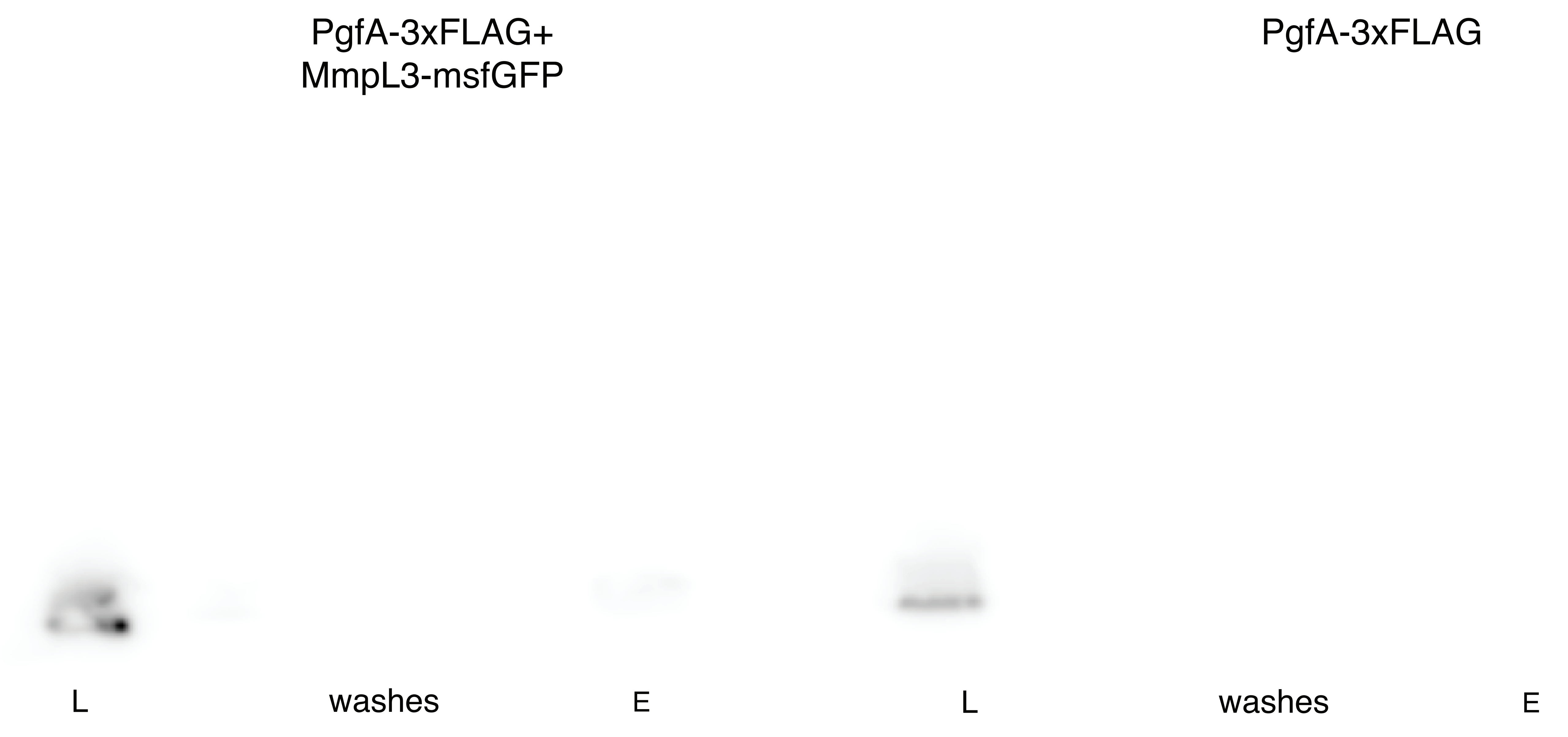
PgfA-3xFLAG+
MmpL3-msfGFP
PgfA-3xFLAG
L
washes
E
L
washes
E
