# Supplementary material for: An essential periplasmic protein coordinates lipid trafficking and is required for asymmetric polar growth in mycobacteria
Source: eLife. 2022 Nov 8;11:e80395. doi: 10.7554/eLife.80395 (PMC9678360; doi:10.7554/eLife.80395)
Supplement: Figure 3—figure supplement 3—source data 4. [file Figure 3 - figure supplement 3 - source data 4.pdf › Figure 3 - figure supplement 3 - source data 4.pdf]

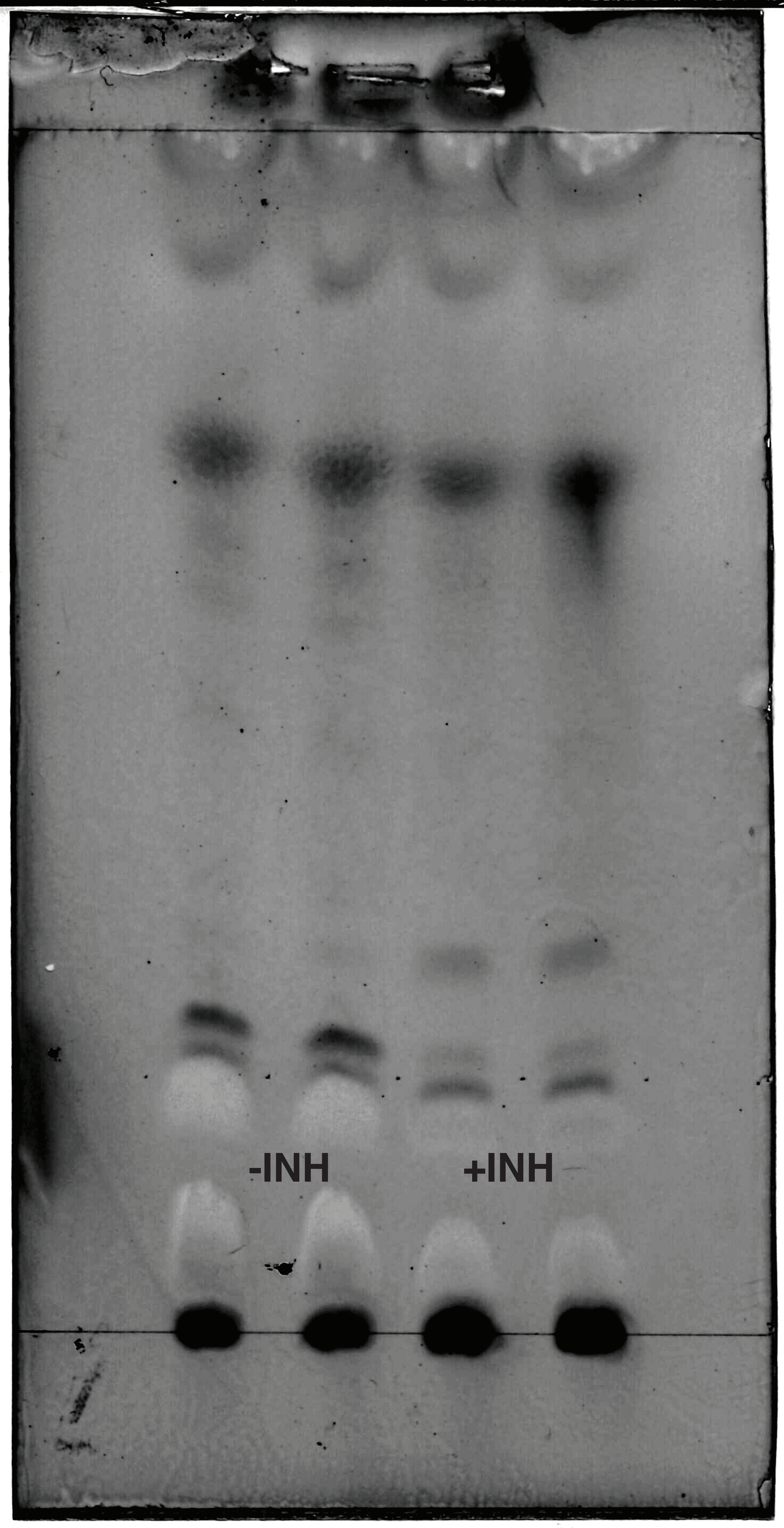
-INH
+INH
